# Differentially Expressed Genes Regulating Glutathione Metabolism, Protein-Folding, and Unfolded Protein Response in Pancreatic β-Cells in Type 2 Diabetes Mellitus

**DOI:** 10.3390/ijms241512059

**Published:** 2023-07-27

**Authors:** Elena Klyosova, Iuliia Azarova, Stepan Buikin, Alexey Polonikov

**Affiliations:** 1 Laboratory of Biochemical Genetics and Metabolomics, Research Institute for Genetic and Molecular Epidemiology, Kursk State Medical University, 18 Yamskaya Street, 305041 Kursk, Russia; klesovaeu@kursksmu.net (E.K.); azzzzar@yandex.ru (I.A.); 2 Department of Biology, Medical Genetics and Ecology, Kursk State Medical University, 3 Karl Marx Street, 305041 Kursk, Russia; 3 Department of Biological Chemistry, Kursk State Medical University, 3 Karl Marx Street, 305041 Kursk, Russia; 4 Centre of Omics Technology, I.M. Sechenov First Moscow State Medical University, 8-2 Trubetskaya Street, 119991 Moscow, Russia; stepan.buikin@novsu.ru; 5 Department of Internal Diseases, Yaroslav the Wise Novgorod State University, 41 Bolshaya St. Petersburg Street, 173003 Veliky Novgorod, Russia; 6 Laboratory of Statistical Genetics and Bioinformatics, Research Institute for Genetic and Molecular Epidemiology, Kursk State Medical University, 18 Yamskaya Street, 305041 Kursk, Russia

**Keywords:** type 2 diabetes, redox homeostasis, glutathione metabolism, β-cells, apoptosis, protein folding, proinsulin misfolding, unfolded protein response, gene expression, differentially expressed genes (DEGs)

## Abstract

Impaired redox homeostasis in the endoplasmic reticulum (ER) may contribute to proinsulin misfolding and thus to activate the unfolded protein response (UPR) and apoptotic pathways, culminating in pancreatic β-cell loss and type 2 diabetes (T2D). The present study was designed to identify differentially expressed genes (DEGs) encoding enzymes for glutathione metabolism and their impact on the expression levels of genes regulating protein folding and UPR in β-cells of T2D patients. The GEO transcriptome datasets of β-cells of diabetics and non-diabetics, GSE20966 and GSE81608, were analyzed for 142 genes of interest using *limma* and GREIN software, respectively. Diabetic β-cells showed dataset-specific patterns of DEGs (FDR ≤ 0.05) implicated in the regulation of glutathione metabolism (*ANPEP, PGD, IDH2,* and *CTH*), protein-folding (*HSP90AB1, HSP90AA1, HSPA1B, HSPA8, BAG3, NDC1, NUP160, RLN1*, and *RPS19BP1*), and unfolded protein response (*CREB3L4, ERP27*, and *BID*). The *GCLC* gene, encoding the catalytic subunit of glutamate–cysteine ligase, the first rate-limiting enzyme of glutathione biosynthesis, was moderately down-regulated in diabetic β-cells from both datasets (*p* ≤ 0.05). Regression analysis established that genes involved in the de novo synthesis of glutathione, *GCLC*, *GCLM*, and *GSS* affect the expression levels of genes encoding molecular chaperones and those involved in the UPR pathway. This study showed for the first time that diabetic β-cells exhibit alterations in the expression of genes regulating glutathione metabolism, protein-folding, and UPR and provided evidence for the molecular crosstalk between impaired redox homeostasis and abnormal protein folding, underlying ER stress in type 2 diabetes.

## 1. Introduction

Diabetes mellitus is one of the fastest-growing global health challenges of the 21st century [1]. Based on the 2021 International Diabetes Federation Diabetes Atlas, 537 million adults aged 20–79 years (a prevalence of 10.5%) are estimated to be living with diabetes mellitus worldwide, and 783 million (a prevalence of 12.2%) are projected to be living with the condition by 2045 [1]. Russia is in second place among all European countries in terms of the number of diabetic patients, 90% of whom are type 2 diabetics (T2D) [1,2]. 

Type 2 diabetes is known as a chronic multifactorial disease characterized by peripheral insulin resistance, impaired hepatic glucose production regulation, and declining pancreatic beta-cell function attributed to cellular death with unknown etiology [3,4]. Dysfunction of pancreatic beta-cell is primary in T2D and is detected at the earliest disease stage, and the loss of beta cells is responsible for disease progression and complications [5,6,7]. It has been observed that oxidative stress in T2D, caused by a shift in redox homeostasis towards decreased antioxidant defense and excessive production of reactive oxygen and nitrogen species, is strongly associated with dysfunction and apoptosis of beta cells, which are extremely sensitive to oxidative damage [8,9]. Endogenous glutathione deficiency has been identified in T2D and is widely agreed to be the primary cause of oxidative stress and mitochondrial dysfunction linked to disease development and progression [10,11,12,13,14,15,16].

In type 2 diabetes, pancreatic beta-cell dysfunction and apoptosis are key processes attributed to the activation of endoplasmic reticulum stress affecting beta cells through PERK–eIF2α-mediated damage [17,18]. We recently hypothesized [16] that glutathione deficiency may be a major cause of impaired folding of proinsulin, which has been identified in type 2 diabetes mellitus [19,20,21]. The hypothesis was based on the assumption that glutathione is essential for regulating the formation of native disulfide bonds within proteins entering the secretory pathway, and numerous studies have demonstrated that glutathione has a strong influence on disulfide pairing in the tertiary structure of target proteins, including proinsulin [22,23,24,25,26,27,28,29,30]. In ensuring protein folding, oxidized glutathione (GSSG) acts as an oxidant, providing disulfide bond formation, whereas reduced glutathione (GSH) functions as a reducing agent, cleaving mis-bridged disulfide bonds in maturating proteins [23,31]. This means that glutathione deficiency, on the one hand, may be per se a key factor responsible for the formation of unfolded or misfolded proteins in the endoplasmic reticulum (ER), on the other hand, contributes to oxidative stress, causing a vicious circle in disrupting disulfide bond formation and aggregation of misfolded proteins, leading to the activation of unfolded protein response and apoptotic pathways in type 2 diabetes. The unfolded protein response (UPR) is a cellular stress response that is activated as a result of the accumulation of unfolded or misfolded proteins in the ER lumen, with the goal of restoring normal cell function by attenuating protein translation, degrading misfolded proteins, and activating signaling pathways that lead to increased production of molecular chaperones that implement protein folding [32]. Numerous studies have demonstrated that chronic ER stress and associated UPR are characteristic features of type 2 diabetes [17,18,33,34,35,36,37]. An intriguing fact is that the accumulation of misfolded proinsulin molecules in the ER due to disruption of the formation of disulfide-related complexes is detected early in prediabetes and subsequently exacerbated, leading to apoptosis and beta-cell dysfunction [19,20,21]. Our recent study discovered that polymorphisms in genes encoding glutathione metabolizing enzymes, such as glutathione synthase (*GSS*) and gamma-glutamyltransferase 7 (*GGT7*), are associated with susceptibility to type 2 diabetes [16]. Other genes involved in glutathione metabolism have also been found to influence the risk of type 2 diabetes [38,39,40,41,42,43]. Interestingly, these polymorphisms were found to correlate with the tissue-specific expression of genes involved in the UPR pathway (*EIF2S2, EIF6, UQCC1, PIGU, ITCH, TRPC4AP, EDEM2, TP53INP2*, and *MAP1LC3A*), glucose-stimulated insulin secretion, and glucose uptake by cells (*MYH7B, CPNE1, TRPC4AP*, and *NCOA6*) [16], providing additional evidence for the existence of shared molecular mechanisms involved in the regulation of glutathione metabolism and protein folding, and linking them to the pathogenesis of type 2 diabetes. No studies have so far been performed to investigate the expression levels of genes regulating protein folding, UPR, and glutathione metabolism in pancreatic beta cells in type 2 diabetes. The objective of this study was to examine the transcriptome profile and co-expression of genes regulating glutathione metabolism, protein-folding, and unfolded protein response in the pancreatic beta cells of type 2 diabetics and non-diabetic individuals.

## 2. Results

### 2.1. Differentially Expressed Genes in Pancreatic Beta Cells in Type 2 Diabetes

We compared the expression levels of three groups of genes: glutathione metabolism, protein folding (molecular chaperones), and unfolding protein response. Table 1 shows differentially expressed genes in the pancreatic β-cells of patients with type 2 diabetes from Dataset 1. As can be seen from Table 1, expression of genes for glutathione metabolism, such as *ANPEP* (*p* = 0.00028) and *G6PD* (*p* = 0.037), was up-regulated in β-cells of T2D patients compared to non-diabetic controls. Meanwhile, *GCLC* (*p* = 0.02) and *PGD* (*p* = 0.02) genes showed a decreased expression in the samples from diabetics as compared with controls. Moreover, a comparative analysis of gene expression between T2D patients and non-diabetics from Dataset 1 showed differences in mRNA levels of eleven genes encoding molecular chaperones and six genes involved in the unfolded protein response pathway. Among genes regulating protein folding, six genes such as *BAG3* (*p* = 0.0003), *HSPA7* (*p* = 0.02), *HSPB2* (*p* = 0.04), *RLN1* (*p* = 0.0018), *NDC1* (*p* = 0.0016), and *TNFRSF21* (*p* = 0.026) were down-regulated, whereas *RPS19BP1* (*p* = 0.0015), *RAE1* (*p* = 0.018), *MTOR* (*p* = 0.018), *POM121C* (*p* = 0.034), and *GFER* (*p* = 0.023) genes were up-regulated in diabetics in comparison with non-diabetic samples. Among genes involved in the unfolded protein response pathway, expression of genes *CREB3L2* (*p* = 0.007)*, CREB3L4* (*p* = 0.0004), and *ERP27* (*p* = 0.0005) was up-regulated, and expression of genes *SSR1* (*p* = 0.008), *DNAJB11* (*p* = 0.035), and *HERPUD1* (*p* = 0.036) was down-regulated in the samples from diabetics as compared with controls. 

Differentially expressed genes in the pancreatic β-cells of T2D patients from Dataset 2 are shown in Table 2. It has been revealed that expression levels of four genes encoding enzymes participating in glutathione metabolism, such as *GCLC* (*p* = 0.05), *PGD* (*p* = 0.002), *IDH1* (*p* = 0.008), and *IDH2* (*p* = 0.006), were decreased in diabetic samples as compared with non-diabetics. In contrast, expression of the *CTH* gene (*p* = 0.003) was up-regulated in β-cells of diabetics compared with non-diabetics. Eleven DEGs have been found among genes involved in protein folding. In particular, decreased expression of molecular chaperones *HSP90AB1* (*p* = 9.8 × 10^−8^)*, HSPA1B* (*p* = 2.8 × 10^−6^)*, HSP90AA1* (*p* = 2.9 × 10^−6^), and *HSPA8* (*p* = 0.001), as well as other genes involved in the regulation of protein folding such as *NUP160* (*p* = 0.002), *ST13* (*p* = 0.05), *POM121C* (*p* = 0.028), and *RPS19BP1* (*p* = 0.005) was observed in pancreatic beta cells of diabetic patients as compared with non-diabetic samples, contrary expressions of three genes encoding nucleoporins *NUP35* (*p* = 0.036), *NUP58* (*p* = 0.026), and *NUP107* (*p* = 0.013) were increased in diabetic samples compared with non-diabetic controls. We also found that five genes involved in the UPR pathway, such as *BID* (*p* = 9.0 × 10^−5^), *DDIT3* (*p* = 0.009), *HYOU1* (*p* = 0.014), *MAPK8* (*p* = 0.016), and *HSPA5* (*p* = 0.028) were down-regulated in beta cells of T2D patients in comparison with non-diabetic subjects. Only the expression of the *EIF2S3* gene (*p* = 0.028) was up-regulated in diabetic samples as compared with non-diabetic samples. Thus, two DEGs regulating glutathione metabolism (*GCLC* and *PGD*) and two DEGs involved in protein folding (*RPS19BP1* and *POM121C*) were found in β-cells of type 2 diabetics from both datasets. However, corrections of the observed P-values for multiple comparisons using the FDR procedure showed that seven DEGs (*ANPEP, BAG3, RLN1, RPS19BP1, NDC1, CREB3L4*, and *ERP27*) in Dataset 1 and ten DEGs (*PGD, IDH2, CTH, HSP90AB1, HSPA1B, HSP90AA1, HSPA8, NUP160, RPS19BP1*, and *BID*) in Dataset 2 reached statistically significant Q-values (FDR < 0.05).

### 2.2. Correlations between Transcription Levels of Differentially Expressed Genes in β-Cells

A correlation analysis was performed to investigate the relationship between expression levels of the studied genes in pancreatic beta cells of T2D patients and non-diabetic controls in both datasets. Correlation matrices of diabetics and controls are shown in Supplementary Table S2. Partial correlations between gene expression levels (*p* ≤ 0.05) are highlighted in red (Supplementary Table S2). Multiple correlations between expression levels genes of glutathione metabolism, molecular chaperons, and unfolding protein response pathway were observed. The correlations between differentially expressed genes in patients with type 2 diabetes and non-diabetics are of interest. 

**Figure 1:**
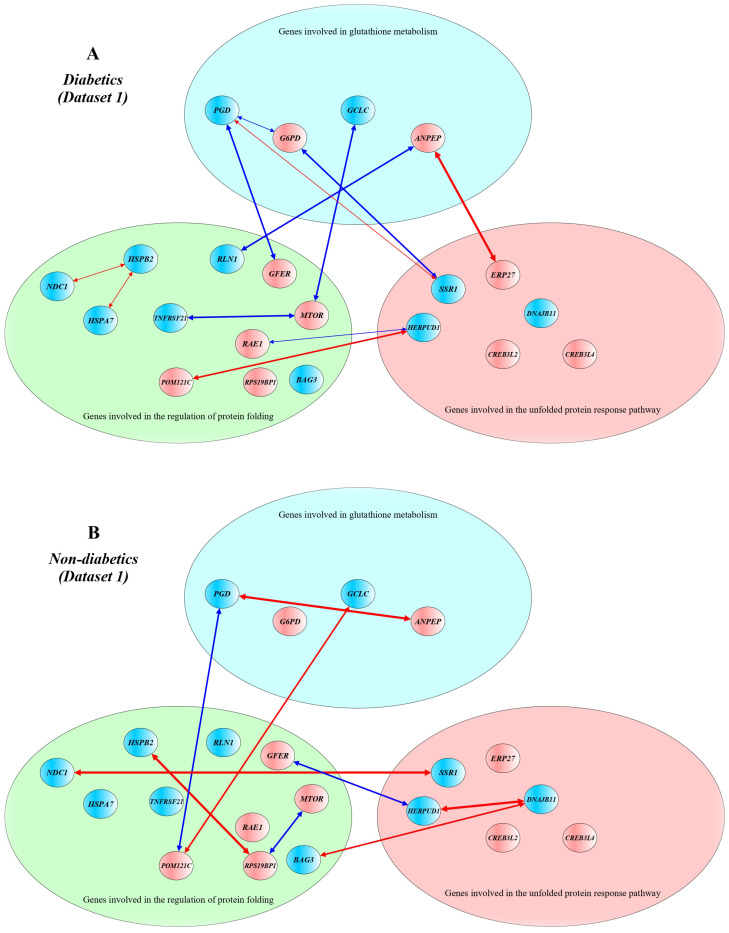
Correlation networks between differentially expressed genes in pancreatic β-cells in patients with type 2 diabetes (**A**) and non-diabetic controls (**B**) from Dataset 1. Networks were constructed using Pearson’s correlations between the DEGs. Up-regulated genes are shown in red, and down-regulated genes are shown in blue. Red lines indicate positive correlations between genes, and blue lines indicate negative correlations between genes. Line thickness reflects the strength of the correlation between genes. A yEd graph editor version 3.22 was used to draw the figure.

Figure 1 shows a schematic presentation of correlations between DEGs in T2D patients (A) and non-diabetics (B) from Dataset 1. In diabetics, *ANPEP* correlated with *ERP27* (r = 0.862) and *RLN1* (r = −0.779), *G6PD* correlated with *PGD* (r = −0.695) and *SSR1* (r = −0.738), *PGD* correlated with *SSR1* (r = 0.677) and *GFER* (r = −0.703), *HSPA7* correlated with *HSPB2* (r = 0.657), *HSPB2* correlated with *NDC1* (r = 0.665), *RAE1* correlated with *HERPUD1* (r = −0.657), *MTOR* correlated with *GCLC* (r = −0.767) and *TNFRSF21* (r = −0.787), *POM121C* correlated with *HERPUD1* (r = 0.727). In non-diabetics, *ANPEP* correlated with *PGD* (r = 0.863), *GCLC* correlated with *POM121C* (r = 0.717), *PGD* correlated with *POM121C* (r = −0.787), *BAG3* correlated with *DNAJB11* (r = 0.796), *HSPB2* correlated with *RPS19BP1* (r = 0.861), *RPS19BP1* correlated with *MTOR* (r = −0.707), *GFER* correlated with *HERPUD1* (r = −0.712), *SSR1* correlated with *NDC1* (r = 0.875), *HERPUD1* correlated with *DNAJB11* (r = 0.874). 

**Figure 2:**
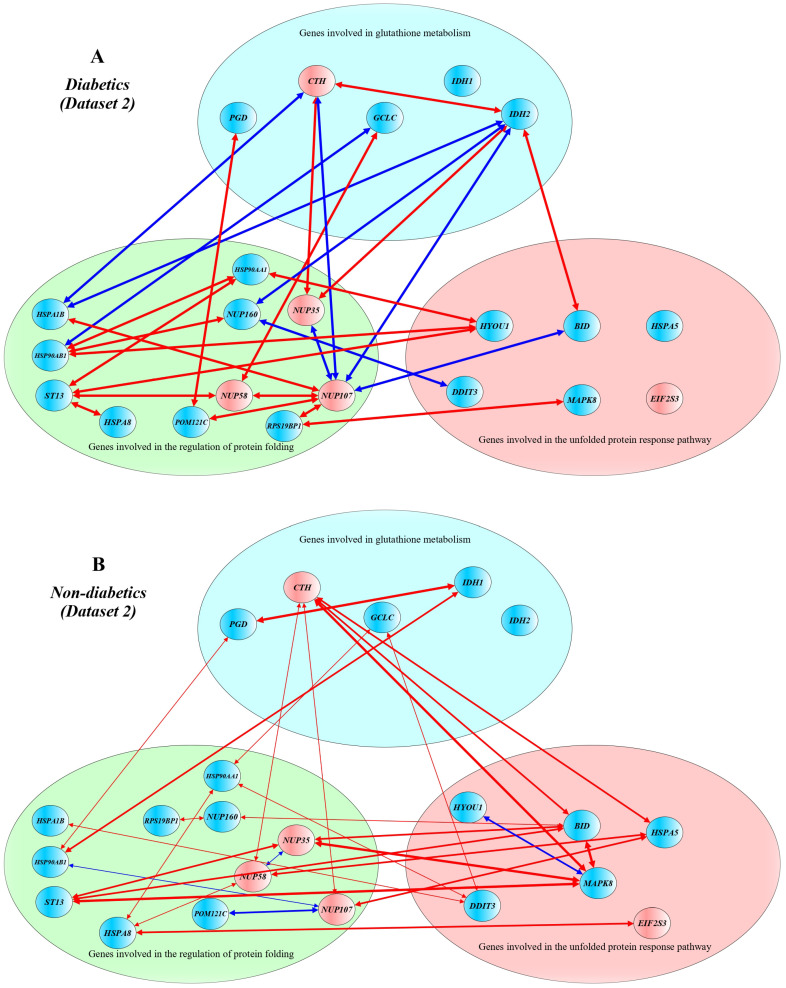
Correlation networks between differentially expressed genes in pancreatic β-cells in patients with type 2 diabetes (**A**) and non-diabetic controls (**B**) from Dataset 2. Networks were constructed using Pearson’s correlations between the DEGs. Up-regulated genes are shown in red, and down-regulated genes are shown in blue. Red lines indicate positive correlations between genes, and blue lines indicate negative correlations between genes. Line thickness reflects the strength of the correlation between genes. A yEd graph editor version 3.22 was used to draw the figure.

Figure 2 shows a schematic presentation of correlations between DEGs in T2D patients (A) and non-diabetics (B) from Dataset 2. In diabetics, *GCLC* correlated with *HSP90AB1* (r = −0.865), *PGD* correlated with *POM121C* (r = 0.832), *IDH2* correlated with *CTH* (r = 0.900), *BID* (r = 0.821), *NUP35* (r = 0.950) and *NUP107* (r = −0.936), *CTH* correlated with *NUP107* (r = −0.905) and *NUP35* (r = 0.835), *HSP90AB1* correlated with *HYOU1* (r = 0.905), *HSP90AA1* (r = 0.983) and *NUP160* (r = 0.868), *HSPA1B* correlated with *CTH* (r = −0.916), *IDH2* (r = −0.878) and *NUP107* (r = 0.900), *HSP90AA1* correlated with *HYOU1* (r = 0.944), *NUP35* correlated with *NUP107* (r = −0.859), *NUP58* correlated with *GCLC* (r = 0.857), *ST13* (r = 0.911) and *NUP107* (r = 0.828), *NUP107* correlated with *POM121C* (r = 0.818), *RPS19BP1* (r = 0.926) and *BID* (r = −0.964), *NUP160* correlated with *IDH2* (r = −0.892) and *DDIT3* (r = −0.895), *ST13* correlated with *HYOU1* (r = 0.851), *HSP90AA1* (r = 0.861) and *HSPA8* (r = 0.864), *RPS19BP1* correlated with *MAPK8* (r = 0.910). In non-diabetics, *GCLC* correlated with *DDIT3* (r = 0.668) and *HSP90AA1* (r = 0.663). *PGD* correlated with *IDH1* (r = 0.818) and *HSP90AB1* (r = 0.641), *HSP90AB1* correlated with *IDH1* (r = 0.750), *CTH* correlated with *BID* (r = 0.782), *HSPA5* (r = 0.731), and *MAPK8* (r = 0.813), *HSP90AB1* correlated with *NUP107* (r = −0.629), *HSPA1B* correlated with *DDIT3* (r = 0.643), *HSP90AA1* correlated with *DDIT3* (r = 0.607), *HSPA8* correlated with *HSP90AA1* (r = 0.686) and *EIF2S3* (r = 0.761), *NUP35* correlated with *BID* (r = 0.792), *MAPK8* (r = 0.862), *NUP58* (r = −0.656) and *ST13* (r = 0.731), *NUP58* correlated with *CTH* (r = 0.627), *HSPA5* (r = 0.776) and *HSPA8* (r = 0.592), *NUP107* correlated with *CTH* (r = 0.637) and *HSPA5* (r = 0.754), *NUP160* correlated with *BID* (r = 0.677) and *RPS19BP1* (r = 0.609), *ST13* correlated with *BID* (r = 0.718), *POM121C* correlated with *NUP107* (r = −0.748), *BID* correlated with *MAPK8* (r = 0.848), *HYOU1* correlated with *MAPK8* (r = −0.784) and *ST13* (r = −0.776), *MAPK8* correlated with *ST13* (r = 0.920). Interestingly, the expression of the insulin gene in β-cells of diabetic patients was positively correlated with the expression of the *CALR* gene in both Datasets 1 (r = 0.884) and 2 (r = 0.953), and this correlation was not observed in non-diabetic samples. Furthermore, in non-diabetic β-cells, insulin expression correlated positively with cyclic AMP-dependent transcription factors involved in the main arms of unfolded protein response: *ATF4* (r = 0.806) in Dataset 1, *ATF3* (r = 0.781) and *ATF6* (r = 0.863) in Dataset 2.

**Figure 3:**
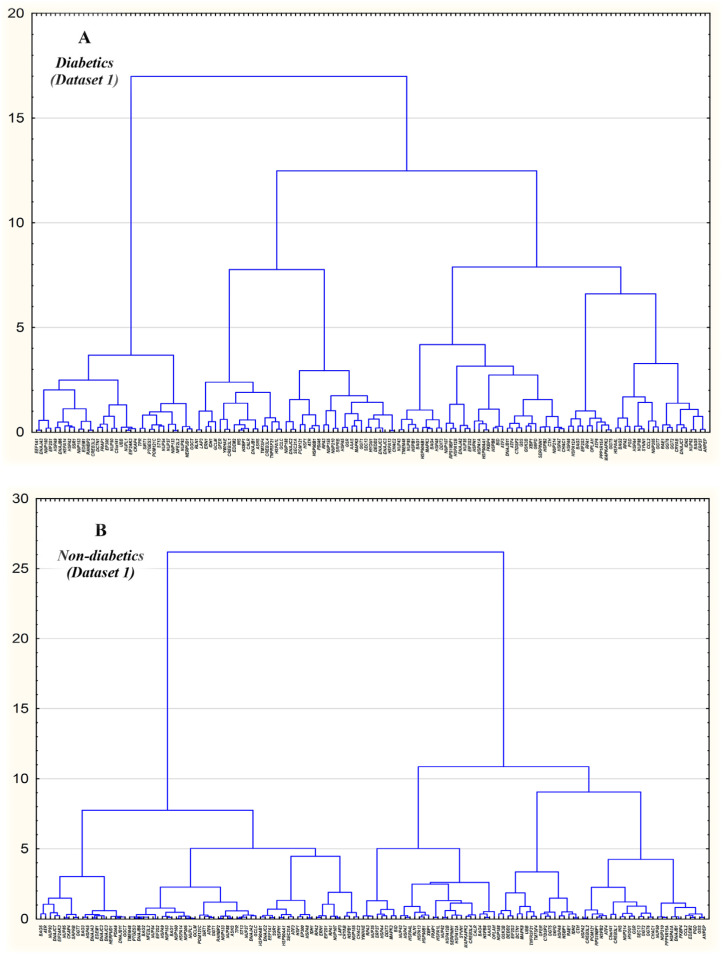
Tree diagrams of correlations between the studied genes in pancreatic β-cells in T2D patients (**A**) and non-diabetic subjects (**B**) from Database 1. Tree diagrams were constructed based on correlation matrices using the hierarchical cluster technique (Ward’s method) in STATISTICA 13.0.

**Figure 4:**
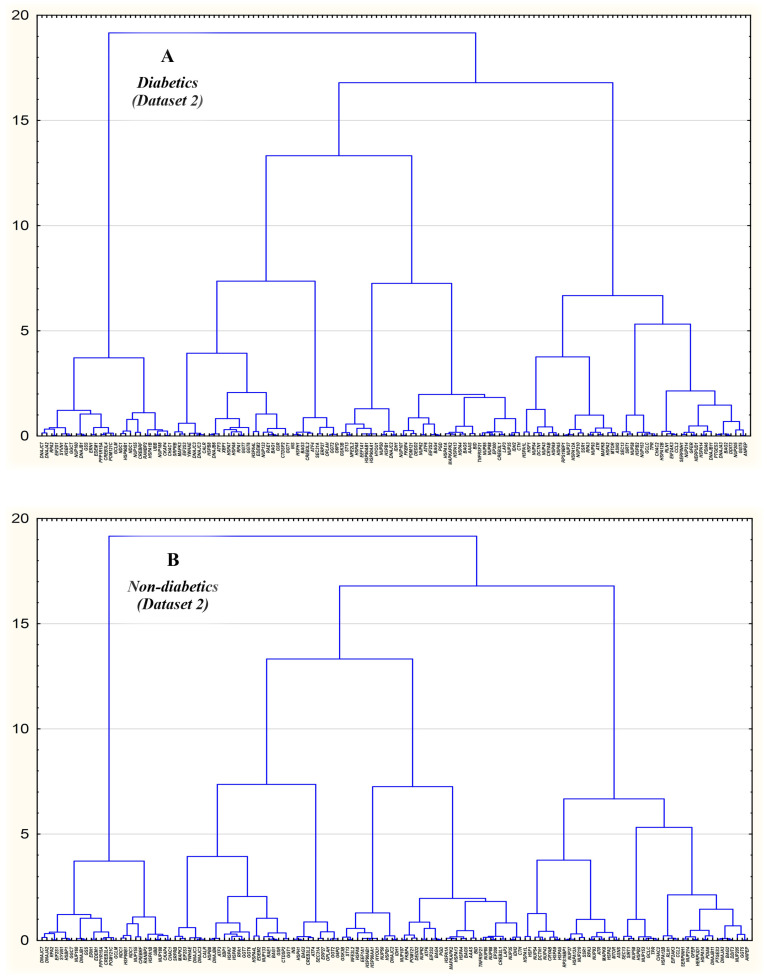
Tree diagrams of correlations between the studied genes in pancreatic β-cells in T2D patients (**A**) and non-diabetic subjects (**B**) from Database 2. Tree diagrams were constructed based on correlation matrices using the hierarchical cluster technique (Ward’s method) in STATISTICA 13.0.

In each dataset, cluster analysis of correlation matrices was used to depict subgroups of inter-correlated genes in diabetics and non-diabetic subjects. Figure 3 and Figure 4 illustrate tree diagrams depicting clusters of inter-correlated expression between genes in T2D patients and non-diabetic controls from Datasets 1 and 2, respectively. Tree networks differed across the T2D samples and datasets. As can be seen from the tree diagram of T2D patients (Figure 3), DEGs such as *ANPEP* and *ERN1*, *G6PD* and *DNAJB11*, and *GCLC* and *TNFRSF21* are present in several independent clusters. In the T2D patients in Dataset 2 (Figure 4), DEGs were also present in independent gene clusters. Interestingly, one such cluster comprised several inter-correlated DEGs such as *HSP90AB1*, *HSP90AA1*, *HYOU1*, *HSPA8*, and *ST13* (*NFE2L2* and *GSK3B* are also present in this cluster). Moreover, *IDH2* and *CTH* genes were united by the shared cluster. The remaining DEGs were present separately in independent clusters.

### 2.3. The Impact of Glutathione Biosynthesis Genes on the Expression Levels of Genes for Protein Folding and Unfolded Protein Response in Pancreatic β-Cells

**Figure 5:**
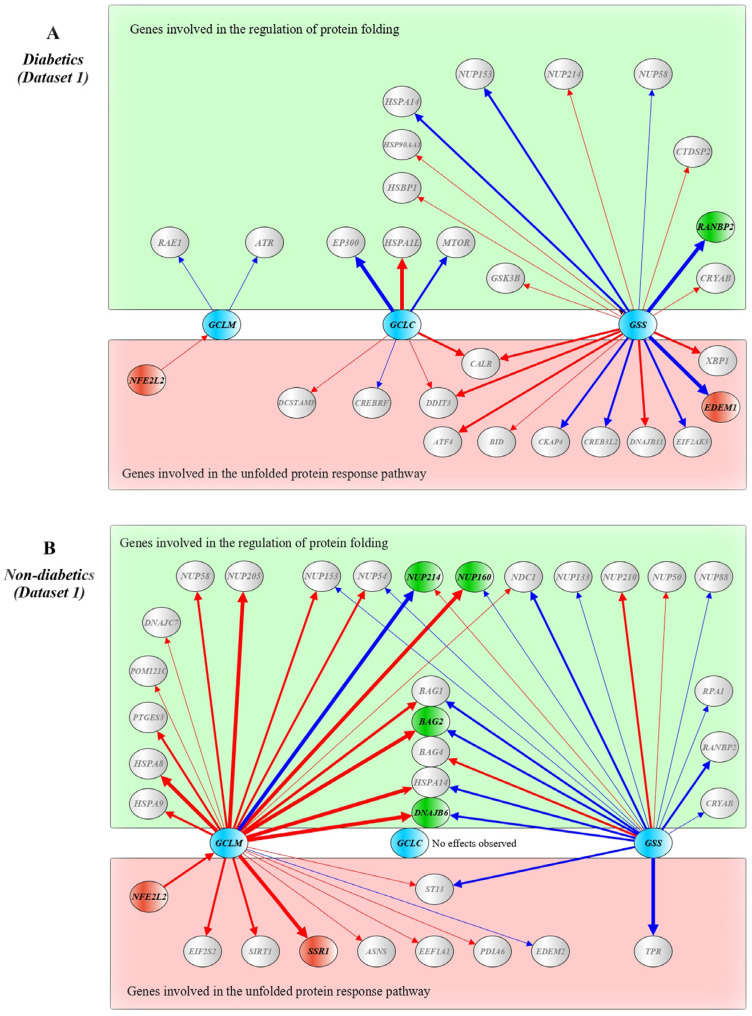
Schematic presentation of the impact of genes encoding enzymes for glutathione biosynthesis on the expression levels of genes for protein folding and unfolded protein response in pancreatic β-cells of diabetic patients (**A**) and non-diabetics (**B**) from Dataset 1. Schemes were constructed using *beta* regression coefficients for the impact of *GCLC*, *GCLM*, and *GSS* genes on the expression levels of genes regulating protein folding and the unfolded protein response. Glutathione biosynthesis genes are shown in blue, genes for protein folding are shown in green, and genes of the UPR pathway are shown in red (the impact on these genes was found at FDR ≤ 0.05; genes which were impacted at *p* ≤ 0.05 and FDR ≥ 0.05 are shown in grey). Red lines indicate positive impacts, and blue lines indicate negative impacts. The line thickness reflects the impact strength. A yEd graph editor version 3.22 was used to draw the figure.

**Figure 6:**
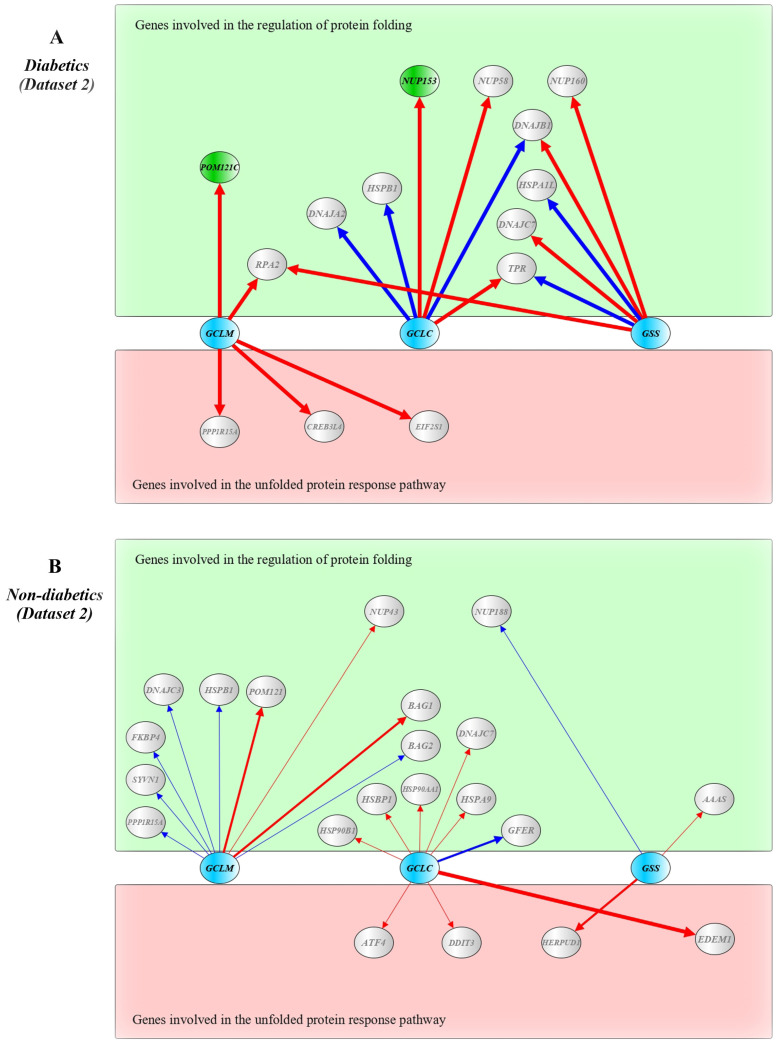
Schematic presentation of the impact of genes encoding enzymes for glutathione biosynthesis on the expression levels of genes for protein folding and unfolded protein response in pancreatic β-cells of diabetic patients (**A**) and non-diabetics (**B**) from Dataset 2. Schemes were constructed using beta regression coefficients for the impact of *GCLC*, *GCLM*, and *GSS* genes on the expression levels of genes regulating protein folding and the unfolded protein response. Glutathione biosynthesis genes are shown in blue, genes for protein folding are shown in green, and genes of the UPR pathway are shown in red (the impact on these genes was found at FDR ≤ 0.05; genes which were impacted at *p* ≤ 0.05 and FDR ≥ 0.05 are shown in grey). Red lines indicate positive impacts, and blue lines indicate negative impacts. The line thickness reflects the impact strength. A yEd graph editor version 3.22 was used to draw the figure.

Since the level of intracellular glutathione is of great importance for the formation of disulfide bonds during the formation of the three-dimensional structure of proteins in the endoplasmic reticulum [23,31], it is very important to investigate whether the expression of genes encoding key enzymes of glutathione biosynthesis, such as *GCLC*, *GCLM*, and *GSS*, affects the transcriptional activity of genes encoding molecular chaperones and regulators of the unfolded protein response. Pursuing this interest, linear regression analysis was applied to investigate the impact of the expression of glutathione biosynthesis genes on the expression levels of genes regulating protein folding and the unfolded protein response in pancreatic beta cells of patients with type 2 diabetes and non-diabetic individuals. The beta regression coefficients estimated for the impact of *GCLC*, *GCLM*, and *GSS* genes on the expression of genes regulating protein folding and the UPR pathway in Datasets 1 and 2 are shown in Supplementary Tables S3 and S4, respectively. A schematic representation of the statistically significant regression coefficients at *p* ≤ 0.05 observed in Datasets 1 and 2 is shown in Figure 5 and Figure 6, respectively. 

In diabetic samples of Dataset 1, *GCLC* influenced the expression levels of *EP300, HSPA1L, MTOR, CALR, DDIT3, CREBRF*, and *DCSTAMP*; *GCLM* influenced the expression of *ATR, NUP153*, and *RAE1*; and *GSS* influenced the expression of *HSPA14, NUP153, NUP214, NUP58, RANBP2, CRYAB, CTDSP2, ATF4, BID, CALR, DDIT3*, *CKAP4, CREB3L2, DNAJB11, EDEM1, EIF2AK3*, and *XBP1*. However, the effects of *GSS* on *RANBP2* (FDR = 0.019) and *EDEM1* (FDR = 0.047) expression remained significant after adjustment for multiple tests. In T2D samples of Dataset 2, *GCLC* influenced the expression levels of *DNAJA2, HSPB1, NUP153, NUP58, TPR*, and *DNAJB1*; *GCLM* influenced the expression of *POM121C, RPA2, CREB3L4, EIF2S1,* and *PPP1R15A*; and *GSS* influenced the expression of *DNAJC7, HSPA1L, NUP160, RPA2, TPR,* and *DNAJB1*. The effects of *GCLC* on *NUP153* (FDR = 0.047) and *GCLM* on *POM121C* (FDR = 0.01). As shown in Supplementary Tables S3 and S4, the effects of *GCLC*, *GCLM*, and *GSS* genes on the expression of genes regulating protein folding and unfolded protein response were observed in pancreatic beta cells of non-diabetics. However, the effects of *GCLM* on the expression of *BAG2, DNAJB6, NUP160, NUP214*, and *SSR1* in samples from Dataset 2 remained significant after adjustment for multiple tests (FDR ≤ 0.05). Interestingly, the impact of the *NFE2L2* gene on the expression of *GCLM* was observed exclusively in non-diabetic β-cells in both Dataset 1 (*beta* = 0.770, *p* = 0.009) and Dataset 2 (*beta* = 0.839, *p* = 0.0006), whereas no such effect was observed in diabetic samples from both datasets.

## 3. Discussion

Despite decades of intensive basic and clinical research, the primary molecular mechanisms underlying the etiology and pathogenesis of type 2 diabetes mellitus remain far from being completed. The question of what causes the loss of pancreatic beta cells, a key pathological process underlying the development and progression of T2D, remains unanswered. It is widely accepted that chronic endoplasmic reticulum stress and activation of the response to unfolded proteins initiate apoptotic pathways that lead to beta-cell death [17,18,19,34,37,44,45]. If the reason for UPR activation in monogenic forms of diabetes is clear—mutations cause structural disorders of the proinsulin molecule, which cannot be folded properly—then, in more common variety type 2 diabetes, the reasons responsible for chronic activation of ER stress remain unknown. We have recently proposed [16] that the deficiency of intracellular glutathione, which is well known to be important in the formation of disulfide bridges in the native tertiary structure of a protein, could be the root cause of proinsulin misfolding in type 2 diabetes. We hypothesize that defective proinsulin folding, a condition that has been linked to type 2 diabetes in numerous studies [19,20,21,46,47,48], may be caused by glutathione deficiency in pancreatic beta cells due to its decreased biosynthesis and/or increased depletion.

It is well known that misfolded or unfolded proteins accumulate in the lumen of the ER due to impaired folding, triggering a cell response to unfolded proteins called the unfolded protein response [32]. UPR is regulated by three ER transmembrane proteins: eukaryotic translation initiation factor 2-alpha kinase 3 (PERK), activating transcription factor 6 (ATF6), and inositol-requiring enzyme 1 (IRE1). PERK, through eIF2α phosphorylation, turns on ATF4, a key transducer that controls the transcription of genes involved in autophagy, apoptosis, amino acid metabolism, and antioxidant responses. ATF6 is transported to the Golgi apparatus, where, as a result of interaction with sites 1 (S1P) and 2 (S2P) proteases, it releases its fragment of the cytosolic domain, ATF6f, which activates genes promoting ER-associated degradation (ERAD) and protein folding. IRE1 and its subsequent auto-transphosphorylation activate the transcription factor XBP1, which controls the transcriptional activity of genes encoding genes involved in protein folding and their quality control as well as ER-associated proteasomal degradation [32,49].

To date, no studies have been conducted to specifically examined the expression of key genes involved in protein folding and the unfolded protein response in pancreatic β-cells of patients with type 2 diabetes. In the present study, we used two independent transcriptomic datasets to look at the expression levels of genes involved in protein folding, UPR, and glutathione metabolism in pancreatic beta cells from diabetic and non-diabetic patients. Compared to non-diabetics, T2D patients were found to have decreased expression of genes regulating protein folding such as *BAG3, NDC1, HSPA7*, *HSPB2, RLN1,* and *TNFRSF21* in Dataset 1, and *HSP90AB1, HSPA1B, HSP90AA1, HSPA8, NUP160, ST13*, and *RPS19BP1* in Dataset 2. Furthermore, several protein folding regulating genes such as *RPS19BP1, RAE1, MTOR, POM121C,* and *GFER* in Dataset 1 and *NUP35*, *NUP58*, and *NUP107* in Dataset 2 have been found to be up-regulated in beta cells from T2D patients when compared to non-diabetics. Induction of chaperones (e.g., from the Hsp70 and Hsp27 families) is known to enhance the adaptive capacity of the endoplasmic reticulum, reduce ER stress, and restore glucose homeostasis, as it has been demonstrated in a mouse model of type 2 diabetes [50]. BAG3 is a member of the BAG family of co-chaperone proteins such as HSP70 and HSC70 [51]. BAG3 is involved in chaperone-assisted selective autophagy, apoptosis, cell adhesion, and cytoskeleton remodeling [52,53,54], suggesting a protective role for this chaperone against type 2 diabetes. Interestingly, the down-regulation of *BAG3* in beta cells was found to increase insulin secretion in response to glucose stimulation [55], and in this context, the decreased levels of *BAG3* mRNA in diabetics may be required for facilitating insulin secretion. No associations of type 2 diabetes with the expression level of heat shock proteins such as *HSPA1B, HSPA7, HSPB2, HSP90AB1, HSPA1B, HSP90AA1,* and *HSPA8* have been reported in the literature. 

*HSPA1B* is a member of the human HSP70 family, whose expression was also increased in the beta cells of diabetics (Dataset 2). Several studies have demonstrated the involvement of the Hsp70 chaperone family in insulin resistance in type 2 diabetes. HSPA1B is a molecular chaperone of the Hsp70 family implicated in a wide variety of cellular processes, including regulation of proteostasis through mechanisms such as folding and transport of newly synthesized polypeptides, quality control of proteins in the ER by ensuring the correct folding of proteins, refolding of misfolded proteins, and controlling the targeting of proteins for subsequent degradation [56,57,58]. Increased serum levels of HSP70 chaperones have been revealed in patients with type 2 diabetes, and they were correlated with disease duration [57]. Polymorphisms in the *HSPA1B* gene have been found to be associated with the severity of diabetic foot ulceration [59] and chronic heart failure [60]. 

HSP90AA1 and HSP90AB1 are members of the Hsp90 family of chaperones comprising 1–2% of cellular protein in unstressed cells and up to 4–6% in stressed cells [61]. Expression of *HSP90AA1* and *HSP90AB1* genes are differentially regulated at the transcriptional level [61]. These chaperones are primarily involved in signal transduction via transcription factors that initiate gene expression, kinases that transmit information by post-translationally modifying other proteins, and E3-ligases that target proteins for proteasomal degradation [61]. It has been hypothesized that biological processes associated with HSP90AB1 are more relevant to maintaining viability, whereas the processes associated with HSP90AA1 are more related to adaptation to stress or other specialized functions [61]. Experiments on mouse models of diabetes have shown that treatment of mice with a pan-Hsp90 inhibitor [62] and also knockdown of HSP90ab1 [63] have been found to improve glucose homeostasis and insulin sensitivity, suggesting a role of these chaperones in the pathogenesis of type diabetes. 

HSPA7 is a putative heat shock 70 kDa protein 7, a pseudogene whose functions are unknown. However, a recent study reported that HSPA7 is a member of long noncoding RNAs (lncRNAs) identified in human atherosclerotic plaques that play a role in the inflammatory transition of vascular smooth muscle cells stimulated by oxidized low-density lipoproteins [64]. HSPA8 (also known as HSC70) is also a member of the Hsp70 family, which, like HSPA1B, is involved in a wide variety of cellular processes such as protection of the proteome from stress, folding and transport of newly synthesized polypeptides, chaperone-mediated autophagy, activation of proteolysis of misfolded proteins and the formation and dissociation of protein complexes [56,57,58]. It has been revealed that patients with type 2 diabetes exhibit higher levels of the stress-related protein complex HSPA8/Hsp90/CSK2α, along with higher platelet aggregation and thrombogenicity than nondiabetic subjects [65]. HSPB2 is a heat shock protein beta-2 which may regulate dystrophia myotonia protein kinase (DMPK) [66]. HSPB2 may also play a role in the regulation of metabolic and mitochondrial functions [67]. It has been experimentally shown that HspB2 deficiency possesses a protective effect form diet-induced glucose intolerance [68].

We also found changes in the expression of nucleoporin genes such as *NDC1, NUP35, NUP58, NUP107, NUP160*, and *POM121C* in pancreatic beta cells of patients with type 2 diabetes mellitus. They are members of the nucleoporin family, structural components of the nuclear pore complex (NPC) [69], which allows for access to the nucleus and controls how proteins and RNA are transported over the nuclear envelope, thereby providing bidirectional molecular movements between the nucleus and cytoplasm [70]. NDC1 (Nuclear-Division-Cycle 1) is a transmembrane nucleoporin (also known as Transmembrane Protein 48 or TMEM48), which plays a key role in de novo assembly and insertion of NPC in the nuclear envelope [69]. Altered expression of NDC1 is observed in ischemic and dilated cardiomyopathy as well as in numerous malignancies [71]. NUP35 also functions as a structural component of NPC, where it plays a role in docking or interacting with partners for transiently associated nuclear transport factors [72]. NUP58 is a nucleoporin, a component of NPC that is involved in nucleocytoplasmic trafficking [73]. NUP107 is a nucleoporin required for the assembly of peripheral proteins into the NPC [74]. NUP160 is a component of NPC involved in the poly (A)+ RNA transport [75]. Interestingly, NUP160 expression was found to be upregulated, which is associated with the inhibition of autophagy and increased inflammatory response in mice with diabetic nephropathy [76]. POM121C is also an essential component of the nuclear pore complex. Interestingly, expression of *POM121C* in beta cells of T2D patients was up-regulated in Dataset 1, and down-regulated in Dataset 2, suggesting its role in diabetes. *POM121C* is considered a candidate gene for fasting serum insulin (FSI), and the FSI-increasing allele at rs1167800 is associated with lower *POM121C* expression, which is associated with systemic insulin sensitivity, adipocyte insulin sensitivity, and adipose hyperplasia [77]. POM121C may contribute to insulin resistance in type 2 diabetes by stimulating adipogenesis and increasing the sensitivity of adipocytes to insulin [77]. Thus, as discussed above, changes in the expression levels of most nucleoporins have not been previously established in type 2 diabetes. However, some studies have demonstrated the importance of nucleoporins in the development of diabetes and its complications.

ST13 is an Hsp70-interacting protein that may contribute to the interaction of HSC70 (chaperone HSPA8) with various target proteins (https://www.uniprot.org/, accession number P50502, accessed on 7 July 2023). RLN1 is a member of relaxins, endocrine, and autocrine/paracrine hormones belonging to the insulin gene superfamily (https://www.uniprot.org/, accession number P04808, accessed on 7 July 2023). TNFRSF21 is a tumor necrosis factor receptor superfamily member 21 that can promote apoptosis, possibly through the activation of NF-kappa-B and BAX pathways and by the release of cytochrome c [78]. TNFRSF21 has recently been shown to play a pathogenic role in heart disease in patients with type 2 diabetes [79]. Increased expression of TNFRSF21 is considered a urinary biomarker of diabetic nephropathy progression [80]. RAE1 (ribonucleic acid export 1) is an mRNA export factor involved in nucleocytoplasmic transport by attaching cytoplasmic microribonucleoproteins to the cytoskeleton [81]. MTOR is a serine/threonine protein kinase that controls cellular metabolism, growth, and survival in response to hormones, growth factors, nutrition, energy, and stress signals (https://www.uniprot.org/, accession number P42345, accessed on 7 September 2023). The mechanistic target of rapamycin (mTOR) signaling is an important intracellular pathway that integrates local nutrition and systemic energy status at the organismal and cellular levels. mTOR signaling dysregulation is linked to a variety of diseases, including type 2 diabetes [82]. MTOR is known to regulate the adaptation of beta cells to blood glucose, and dysregulation in mTOR signaling may facilitate the development of type 2 diabetes or insulin resistance. It has been revealed that short-term mTORC1 activation enhances beta cell mass and improves glucose metabolism, whereas long-term mTORC1 activation deteriorates beta cell mass and function, as observed in type 2 diabetic beta cells [82,83]. RPS19BP1 is another gene that showed differential expression in the beta cells of diabetics in both analyzed datasets. RPS19BP1 is a ribosomal protein S19 binding protein 1, which is a part of the small subunit processome that directly regulates SIRT1 [84]. It has been demonstrated experimentally in mice that the activation of SirT1 optimizes the organism for metabolic adaptation to insulin resistance by improving hepatic insulin sensitivity and decreasing whole-body energy requirements [85]. This finding suggests that RPS19BP1-mediated activation of SIRT1 plays a role in the pathogenesis of type 2 diabetes. Increased expression of *GFER* in pancreatic beta cells in T2D patients was found in Dataset 1. The *GFER* gene (growth factor, augmenter of liver regeneration) encodes a protein, a part of the CHCHD4:GFER complex, also known as a disulfide relay machinery, which catalyzes the oxidation of cysteine residues in precursor proteins in the mitochondrial intermembrane space to form disulfide bonds, determining protein folding and stability [86,87,88]. *GFER* maintains mitochondrial ROS levels for optimal functioning of complexes III and IV of the electron transport chain through glutathionation and promotes the formation of disulfide bonds in the CHCHD4 chaperone molecule, and polymorphism of the *GFER* has been found to be associated with the risk of type 2 diabetes [89].

Genes regulating the unfolded protein response that are differentially expressed in the beta cells of patients with type 2 diabetes are of great interest. In particular, In particular, *CREB3L2, CREB3L4*, and *ERP27* were up-regulated, whereas *SSR1, DNAJB11,* and *HERPUD1* were down-regulated in pancreatic β cells in diabetics when analyzed in Dataset 1. In Dataset 2, decreased expression of *BID, DDIT3, HYOU1, MAPK8*, and *HSPA5*, along with increased expression of EIF2S3, were found in T2D samples compared to non-diabetic controls. CREB3L2 and CREB3L4 are ER-localized proteins belonging to the bZIP family and are transcriptional activators that play roles in the unfolded protein response as regulators of cell secretory capacity and cell-specific cargo [90]. Importantly, transcription factors of the CREB3 family were found to regulate high-fat diet-induced obesity and energy metabolism, which are conditions associated with type 2 diabetes [91]. It has been revealed that Creb3l4 knockout mice exhibit glucose tolerance and decreased insulin sensitivity [92], suggesting a role of CREB3L4 in both obesity and type 2 diabetes. ERP27 (a noncatalytic member of the protein disulfide isomerase) is an endoplasmic reticulum-resident protein that plays a role in the unfolded stress response by binding to unfolded proteins and recruiting PDIA3 protein disulfide isomerase to unfolded substrates (ERP27 has a unique affinity for unfolded proteins and may attract protein disulfide I to substrates that are unfolded) [93] and expression of *ERP27* in beta cells was up-regulated in diabetic patients [94]. EIF2S3 (eukaryotic translation initiation factor 2 subunit 3) is an eIF2 complex component that functions in the early stages of protein synthesis by building a ternary complex with GTP and an initiator tRNA (https://www.uniprot.org/, accession number P41091, accessed on 7 October 2023). EIF2S3 may play a critical role in human hypothalamo-pituitary development and function and regulation of glucose metabolism [95], and mutations in the *EIF2S3* gene may contribute to neonatal hypoglycemia, followed by early onset diabetes and hypopituitarism [96]. The *SSR1* (signal sequence receptor subunit 1) gene encodes translocon-associated protein (TRAP) subunit alpha, being a part of a complex that binds calcium to the ER membrane and thereby regulating the retention of ER-resident proteins and may function as a membrane-bound chaperone facilitating folding of translocated proteins (https://www.uniprot.org/, accession number P43307, accessed on 7 October 2023). It is important to note that common single-nucleotide polymorphisms in the human TRAP gene are linked to type 2 diabetes susceptibility, and the associated pancreatic cell dysfunction indicates that impaired preproinsulin translocation and proinsulin trafficking may play a role in T2D pathogenesis [97]. HSPA5 is an endoplasmic reticulum chaperone BiP with a variety of biological functions, including a key role in protein folding and quality control in the ER lumen [98], post-translational transport of small presecretory proteins across the ER [99], provides the correct folding of proteins, and degradation of misfolded proteins [100], and acts as a key repressor of the ERN1/IRE1-mediated arm of the UPR pathway [100]. HSPA5 is also known as GRP78, whose serum concentration was found to be significantly higher in T2D patients than in healthy controls and was positively correlated with HbA1c levels in diabetics [101]. Experiments in mice suggest that decreased GRP78 expression in the liver may induce resistance to insulin by inhibiting AKT activation and may play an important role in the development of type 2 diabetes [102]. The overproduction of GRP78 in pancreatic beta cells was found to protect mice against high-fat diet-induced diabetes [103]. DNAJB11 is known as a co-chaperone for HSPA5 and is required for the proper folding, trafficking, or degradation of proteins [104]. DNAJB11 binds to unfolded proteins, which are ERAD substrates, as well as to nascent unfolded peptide chains [105]. This co-chaperone may help recruit HSPA5 and other chaperones to the substrate and stimulate the ATPase activity of HSPA5 [106]. Notably, decreased expression of DNAJB1 in pancreatic islets has also been observed in mice with experimental T2D [107]. HERPUD1 (homocysteine inducible ER protein with ubiquitin-like domain 1) is a component of the ER quality control system involved in ubiquitin-dependent ERAD degradation of misfolded proteins [108]. Herpud1 has been found to regulate insulin secretion in mice [109]. BID (BH3 interacting domain death agonist (BID) is a protein that induces caspase and apoptosis pathways [110] and allows the release of cytochrome c by mitochondria [111]. An experimental study on mouse islets demonstrated that Bid is essential for death receptor-induced apoptosis of islets [112], suggesting its role in beta cell dysfunction in type 2 diabetes. DDIT3 (DNA damage-inducible transcript 3 protein), also known as CHOP, represents a multifunctional transcription factor playing an essential role in response to a variety of cell stresses and induces cell cycle arrest and apoptosis in response to ER stress [113,114]. It has been revealed that depletion of *Ddit3* in β cells alleviates ER stress in mice [115]. Similar results were obtained in a study by Oyadomari with colleagues [116]. HYOU1 is a hypoxia-upregulated protein 1, ER chaperone that plays a key role in cytoprotection under oxygen deprivation and also as a molecular chaperone participating in protein folding [117]. Up-regulation of *HYOU1* and *HSPA5* was found in the tubular epithelia of patients with diabetic nephropathy compared with control kidneys [118]. MAPK8 is a mitogen-activated protein kinase 8 that is responsive to various cellular stress stimuli and regulates a wide range of biological processes, including cellular proliferation, differentiation, migration, transformation, autophagy, and apoptosis [119]. Like other MAPKs, MAPK8 may affect insulin signaling and, therefore, play a role in the pathophysiology of type 2 diabetes [120,121]. Moreover, MAPKs have been observed to phosphorylate the HSF1 chaperone, inhibiting HSF1-induced transcription [122], which may result in decreased downstream chaperone activation and protein folding. A decrease in *MAPK8* gene expression in type 2 diabetes pancreatic beta cells could be due to, on the one hand, a shift in insulin signaling and glucose transport into the cell and, on the other hand, a decrease in chaperone activation and protein folding. Undoubtedly, all the above assumptions require experimental confirmation. Thus, the results obtained from the analysis of both datasets, despite the observation of dataset-specific DEGs, showed that in pancreatic beta cells, there were changes in the expression of genes that regulate protein folding and those involved in the regulation of UPR. These findings are in line with those of studies that observed abnormal folding of proinsulin in patients with type 2 diabetes [19,20,21]. 

Given the relevance of defective glutathione metabolism in the pathophysiology of type 2 diabetes [10,11,12,15,16], genes encoding enzymes involved in glutathione metabolism regulation may contribute to disease development and are attractive targets for diabetes research. This sparked an interest in studying the expression levels of these genes in pancreatic beta cells from type 2 diabetes patients. We also wanted to know if the expression levels of glutathione metabolism genes are related to the expression levels of genes that govern protein folding and those involved in the unfolded protein response. The current study identified for the first time differentially expressed genes involved in glutathione metabolism in type 2 diabetes pancreatic beta cells. In particular, we found a decreased expression of the *GCLC* gene in the samples from patients with type 2 diabetes compared with non-diabetic controls in both datasets. The *GCLC* gene encodes the catalytic subunit of glutamate–cysteine ligase, the first-rate limiting enzyme of glutathione biosynthesis [123], and therefore the decreased expression of *GCLC* in T2D patients may contribute to a diminished capacity of beta cells to synthesize glutathione. This assumption may be supported by our recent findings that genetic variants in glutamate–cysteine ligase confer protection against type 2 diabetes due to their positive effect on glutathione levels [38]. Moreover, the levels of *ANPEP* were increased in the beta cells of diabetics as compared with non-diabetic samples, as has been reported previously by Marselli and co-authors [94] and further replicated in a study by Locke and colleagues [124]. Interestingly, transforming growth factor-beta1, whose plasma concentrations are increased in type 2 diabetes and diabetic renal disease [125,126], was found to increase both the expression and activity of ANPEP in a time- and concentration-dependent manner [127]. Moreover, polymorphisms of the *ANPEP* gene were found to be associated with the risk of T2D [128,129]. Thus, the above findings clearly show that the *ANPEP* gene can be involved in the pathogenesis of type 2 diabetes. The mechanisms by which ANPEP contributes to T2D susceptibility remain unclear. ANPEP is alanyl aminopeptidase, an enzyme with broad substrate specificity catalyzing the hydrolysis of peptide bonds with the removal of amino acids from the amino terminus of proteins and peptides. It is important to note ANPEP hydrolyzes peptide L-cysteinylglycine to cysteine and glycine—substrates for de novo biosynthesis of GSH [130,131]. As we recently proposed [16] that the increased levels of *ANPEP* mRNA in pancreatic beta cells and plasma levels of GGT1 (gamma-glutamyltransferase) in T2D patients may be indicative of an adaptive mechanism in response to endogenous glutathione deficiency: up-regulation of these membrane-bound enzymes in diabetes is necessary for the sequential production of three amino acids (AA)—precursors for intracellular GSH biosynthesis. In particular, GGT1 catabolizes extracellular GSH into L-glutamate (first AA) and L-cysteinylglycine [100,101], then the latter is hydrolyzed by ANPEP with the formation of cysteine (second AA) and glycine (third AA) [130,131], which are then transported into the cell for GSH biosynthesis. This hypothesis is attractive in that it explains several seemingly unrelated biochemical abnormalities that are often detected in type 2 diabetes mellitus and, at the same time, explains the commonality of their occurrence as a result of an endogenous deficiency of glutathione.

In addition, we observed decreased levels of *PGD* in pancreatic beta cells of diabetic patients compared with non-diabetic controls in both datasets. Furthermore, decreased expression of *IDH1* and *IDH2*, increased expression of *CTH* in Dataset 2, and increased expression of *G6PD* in Dataset 1 were observed in T2D samples, in contrast to the controls. The *PGD* gene encodes phosphogluconate dehydrogenase, an enzyme catalyzing the oxidative decarboxylation of 6-phosphogluconate to ribulose 5-phosphate and CO_2_, a reaction accompanying the reduction of NADP to NADPH (https://www.uniprot.org/, accession number P52209, accessed on 7 October 2023). IDH1 (isocitrate dehydrogenase (NADP) cytoplasmic) is an enzyme that catalyzes the NADP+-dependent oxidative decarboxylation of isocitrate to 2-ketoglutarate with generation NADPH [132]. IDH2 is a mitochondrial isocitrate dehydrogenase (NADP), an enzyme associated with the pyruvate dehydrogenase complex and a major source of NADPH, playing a role in energy production [133]. Knockdown of the mitochondrial isocitrate dehydrogenase enzyme in pancreatic beta cells inhibits insulin secretion [134]. Interestingly, transcriptional up-regulation of *IDH2* expression has been found to be associated with improved mitochondrial function in endothelial progenitor cells in diabetic patients [135]. A recent study observed that elevated levels of IDH2 in the mouse liver under overnutrition contribute to elevated gluconeogenesis and glycogen synthesis [136]. Thus, it can be assumed that deficiency of IDH1 and IDH2 in pancreatic beta cells may be associated with a decrease in the formation of G6PD, which is necessary for the reduction of GSSG to GSH. G6PD (glucose-6-phosphate dehydrogenase) is an enzyme of the pentose–phosphate shunt, representing an alternative to the glycolysis pathway in the dissimilation of carbohydrates, with the primary function to supply NADPH for many biosynthesis pathways, including the reduction of oxidized glutathione [137,138]. A systematic review and meta-analysis showed that G6PD deficiency may be a risk factor for diabetes [139]. We can hypothesize that increased expression of *G6PD* in beta cells may favor the production of NADPH, which is necessary for the reduction of oxidized glutathione. CTH (cystathionine gamma-lyase) is an enzyme that catalyzes the last step in the transsulfuration pathway from L-methionine to L-cysteine, which is utilized for glutathione biosynthesis [140]. CTH, an enzyme of the transsulfuration pathway supplying cysteine for GSH synthesis, whose expression level in beta cells in T2D patients was higher than that in non-diabetic controls (dataset 2). CTH is known to promote PERK activity, leading to attenuation of protein translation during the response to ER stress [141]. It can be assumed that the increased expression of the *CTH*, *ANPEP*, and *G6PD* genes in T2D samples compared with non-diabetic ones appear to be adaptive and aimed at replenishing glutathione deficiency in beta cells of diabetics: CTH and ANPEP supply amino acids such as L-cysteine (CTH and ANPEP) and glycine (ANPEP) for de novo biosynthesis of GSH, whereas G6PD generates NADPH for the reduction of GSSG to GSH. The correlations between genes involved in glutathione metabolism confirm the preceding assumptions about potential disruptions in glutathione synthesis in pancreatic beta cells in type 2 diabetes (Figure 1 and Figure 2). 

We also found correlations in expression level between genes for glutathione metabolism and genes regulating protein folding and unfolded protein response. In particular, in T2D patients from Dataset 1, *ANPEP* correlated with *ERP27* and *RLN1*, *G6PD* correlated with *PGD* and *SSR1*, *PGD* correlated with *SSR1* and *GFER*, *HSPA7* correlated with *HSPB2*, *HSPB2* correlated with *NDC1*, *RAE1* correlated with *HERPUD1*, *MTOR* correlated with *GCLC* and *TNFRSF21*, *POM121C* correlated with *HERPUD1*. In diabetics from Dataset 2, *GCLC* correlated with *HSP90AB1*, *PGD* correlated with *POM121C*, *IDH2* correlated with *CTH*, *BID*, *NUP35* and *NUP107*, *CTH* correlated with *NUP107* and *NUP35*, *HSP90AB1* correlated with *HYOU1*, *HSP90AA1* and *NUP160*, *HSPA1B* correlated with *CTH*, *IDH2* and *NUP107*, *HSP90AA1* correlated with *HYOU1*, *NUP35* correlated with *NUP107*, *NUP58* correlated with *GCLC*, *ST13* and *NUP107*, *NUP107* correlated with *POM121C*, *RPS19BP1* and *BID*, *NUP160* correlated with *IDH2* and *DDIT3*, *ST13* correlated with *HYOU1*, *HSP90AA1* and *HSPA8*, *RPS19BP1* correlated with *MAPK8*. An interesting finding was seen in both datasets: the expression of the insulin gene in β-cells of T2D patients positively correlated with the expression of CALR, a calcium-binding chaperone that promotes folding, oligomeric assembly, and quality control in the ER (https://www.uniprot.org/, accession number P27797, accessed on 7 December 2023), and this cor-relation was not observed in non-diabetic samples. Furthermore, in non-diabetic β-cells, insulin expression correlated positively with cyclic AMP-dependent transcription factors involved in the main arms of the unfolded protein response: ATF4 in Dataset 1 and ATF3 and ATF6 in Dataset 2. These findings suggest that the expression of insulin in type 2 diabetes is co-regulated by gene networks governing protein folding and the unfolded protein response in beta cells. Cluster analysis revealed groups of inter-correlated genes, demonstrating that the differentially expressed genes in beta cells in type 2 diabetes may have a relatively independent regulation. For instance, *ANPEP* showed a positive correlation with *ERP27* (both genes were strongly up-regulated in T2D samples), and these genes were united by a shared relatively independent gene cluster (Figure 3A).

Taken together, these findings suggest co-expression of genes regulating glutathione metabolism and genes involved in protein folding and the UPR pathway. Such correlations were expected because it is known that glutathione is required to regulate the formation of native disulfide bonds within proteins entering the secretory pathway [22,23,142]; therefore, cross-talk between glutathione and protein folding pathways is native. It was not surprising that correlations in gene expression levels differed in Datasets 1 and 2. We hypothesize that differences in gene signatures in beta cells of diabetics between the datasets may be attributed to a variety of reasons, including the degree of glutathione deficiency determining the redox state due to insufficient intake of amino acid GSH precursors into the cell, the impact of polymorphisms at genes encoding enzymes of glutathione metabolism, and genes whose products process protein folding in the endoplasmic reticulum and regulate the unfolded protein response.

The unfolded protein response is a universal molecular mechanism that maintains proteostasis under stress in both the endoplasmic reticulum and mitochondria, which are the primary energy metabolism and protein biosynthesis centers in cells [36]. In type 2 diabetes, dysregulated UPR signaling occurs in both the endoplasmic reticulum and the mitochondria, which are considered to crosstalk via the PERK signaling pathway [36]. In pancreatic β-cells, mitochondrial activity is essential for glucose-stimulated insulin secretion and mitochondrial dysfunction to the pathophysiology of type 2 diabetes [143]. According to a recent study, mitochondrial metabolic activity is communicated to the ER via the relay of redox metabolites, and increasing in the oxidized glutathione may influence oxidative protein maturation and protect from ER stress [138]. Glutathione is known to maintain the proper mitochondrial redox environment by acting directly or as a cofactor in reactions catalyzed by other mitochondrial enzymes, as well as preventing oxidative modifications that can lead to mitochondrial dysfunction [144]. Given the foregoing, it is reasonable to assume that metabolic pathways like glutathione metabolism, protein folding, and unfolded protein response are coordinately regulated and that identifying these common pathways will allow us to better understand the nature of metabolic disorders underlying type 2 diabetes. Biological processes underpinning the link between glutathione metabolism, protein folding, and cellular response to unfolded proteins are subjects of great interest. First of all, since glutathione provides the main redox buffer against ER-generated oxidative stress by maintaining ER oxidoreductases in a reduced state, it has been implicated in the formation of native disulfide bonds in proteins in the endoplasmic reticulum through a complex process involving not only disulfide-bond formation but also the isomerization of non-native disulfide bonds [22]. Protein disulfide isomerase (PDI) is capable of catalyzing both processes [145]. For disulfide bond formation, the ratio of reduced to oxidized glutathione ((GSH):(GSSG)) is optimal [146,147]. Disulfide-bond formation in the ER can lead to the formation of ROS, and their detoxification by GSH results in an increase in GSSG [22]. Notably, increased ROS generation is linked to protein misfolding [148,149]. Decreased total glutathione levels were found to be associated with increased formation of disulfide bonds, which were simultaneously accompanied by increased formation of non-native disulfide bonds [22]. These findings could be explained by the fact that PDI’s ability to isomerize non-native disulfide bonds is limited as this enzyme becomes more oxidized. Thus, it could be suggested that the elevated levels of glutathione in the ER are required for both the elimination of ROS and the facilitation of native disulfide-bond formation in folding proteins. This means that a deficiency of glutathione in the cell can disrupt these processes, causing, on the one hand, oxidative stress and, on the other hand, disruption of protein folding with subsequent activation of the UPR pathway. Notably, activation of unfolded protein response has been found to be associated with an increase in glutathione biosynthesis. It has been observed that the ATF4 and Nrf2 transcription factors link ER stress to a broader cellular response that boosts glutathione synthesis via enhancing amino acid metabolism [150,151]. Transcription factor Nrf2 plays a key role in the response to oxidative stress of the NFE2L2/NRF2 pathway [152]. Interestingly, the positive impact of *NFE2L2* (gene encoding Nrf2) on the expression of the glutamate–cysteine ligase modifier subunit (GCLM) was observed only in the β-cells of individuals without type 2 diabetes, whereas no such effect was observed in diabetic samples from both datasets. This finding suggests that the NFE2L2/NRF2 pathway does not activate glutathione biosynthesis in the beta cells of patients with diabetes. The activation of PERK results in a general decrease in protein synthesis and an increase in glutathione synthesis [22]. Moreover, oxidized glutathione may enhance chaperone activity, as it has been demonstrated for the folding of alpha-crystallin [153]. Taken together, these data clearly indicate that optimal levels of glutathione are vitally important in endoplasmic reticulum for efficient protein folding and protection against oxidative stress.

Glutathione is necessary for the formation of disulfide bonds in proteins, the process in which ROS are produced as by-products, leading to impaired redox homeostasis in the ER and oxidative stress [154,155], a pathological condition prevented by glutathione. ROS have been shown to activate the UPR, which can be reversed by N-acetylcysteine by replenishing the glutathione pool. It was shown that oxidative stress may explain the activation of ER stress [154]. This means that protein folding in the ER is dependent on redox homeostasis, and oxidative stress can disrupt the mechanisms of effective protein folding, enhancing the production of misfolded or unfolded proteins and causing ER stress. Given glutathione’s critical role in maintaining redox homeostasis and ensuring the process of protein folding in the endoplasmic reticulum, there is reason to believe that glutathione deficiency and the associated defective ER folding of proteins may be the cause of the increased amount of misfolded proinsulin molecules found in type 2 diabetes. Due to the fact that pancreatic beta cells have extremely high protein-synthetic activity (they produce almost 1 million insulin molecules per minute [156]), this ability makes the pancreas extremely sensitive to ER stress. Studies of type 2 diabetes mellitus show that the process of proinsulin folding is closely related to the regulation of ER stress and unfolded protein response, whose prolonged activation triggers the apoptosis of pancreatic beta cells [44].

The study has several limitations. Firstly, the range of selected genes involved in protein folding and unfolded proteins is incomplete since we have selected the main genes presented simultaneously in three metabolic databases such as KEGG, Reactome, and WikiPathways. For example, we did not look at many genes that encode enzymes from the protein disulfide isomerase gene family, which is important in enzyme-mediated disulfide bond formation in proteins. In this regard, it cannot be ruled out that alterations in the expression of genes not studied in the current study may exist. Secondly, the transcriptome data samples of pancreatic beta cells from T2D patients and non-diabetic controls used in the study were small and characterized by significant variability in gene expression across samples. Third, the comparison results obtained from the transcriptome datasets generated using different methodologies resulted in different scales of gene expression, thereby neutralizing similar trends in changes in gene expression in diabetes between the datasets. This means that establishing alterations in gene expression in type 2 diabetes relative to controls in both datasets was extremely challenging. Fourth, when interpreting the results, one should also take into account the fact that differentially expressed genes may reflect disease-induced rather than disease-causing changes in the transcriptome, as was noted by Porcu with co-workers [157]. In addition, it cannot be ruled out that changes in gene expression may be associated with other pathological processes present in patients. Furthermore, the correlations between expression levels of the studied genes should be interpreted with caution since they were inflated due to the small number of investigated pancreatic β-cell samples. Further studies on larger samples obtained at an early stage of the disease, as well as the use of unified methodological techniques for gene expression analysis, would allow a more objective assessment of the nature of gene expression changes in pancreatic beta cells in type 2 diabetes.

## 4. Materials and Methods

### 4.1. Datasets

Gene Expression Omnibus (GEO) datasets on gene expression profiles in pancreatic islets of Langerhans from type 2 diabetes patients and non-diabetic subjects were downloaded from the GEO database (https://www.ncbi.nlm.nih.gov/geo/ (accessed on 14 February 2022)). The keywords ‘type 2 diabetes’ (All Fields) AND ‘Homo sapiens’ (Organism) AND ‘beta cells’ (All Fields) OR ‘islet cells’ (All Fields) OR ‘pancreatic cells’ (All Fields) were used to search for gene expression datasets. The list of found datasets was manually evaluated to identify datasets with transcriptome profiles of islet beta cells from T2D patients and non-diabetic controls. As a result, two datasets, GSE20966 (Dataset 1) and GSE81608 (Dataset 2), with transcriptomic profiles of pancreatic islets from patients with type 2 diabetes and those without diabetes, were identified and selected for the present study.

Dataset 1 includes gene expression profiles of beta cells obtained by laser capture microdissection from cadaver pancreases of 10 subjects with T2D and 10 non-diabetics of European descent [94]. Extracted RNA samples were amplified, biotinylated, and hybridized to the GeneChip Human X3P Array (Affymetrix, Santa Clara, CA, USA). Dataset 2 represents the transcriptomes of 1492 single human pancreatic islet cells such as α-, β-, δ- and pancreatic polypeptide (PP cells) that were obtained from cadaveric pancreas of 6 T2D and 12 non-diabetics (70% were Europeans, 30% were Asians or Hispanics) [158]. Pancreatic islet cell types were hybridized with mRNA probes for human GCG, INS, SST, and PPY genes to estimate bimodal expression distribution of the genes. Isolated RNA samples from each cell type were analyzed using single-cell RNA sequencing on Illumina HiSeq2500 (Illumina, San Diego, CA, USA). RNA-Seq data analysis was performed after the quality control of gene expression. Expressed genes were defined by ≥ 1 RPKM. The transcriptome data of single cells (β-cells) obtained from each donor were averaged.

### 4.2. Selection of Genes

The following groups of genes or pathways were subjects of interest for our study: (1) genes encoding molecular chaperones or those implementing protein folding; (2) genes involved in the unfolded protein response (UPR); and (3) genes encoding enzymes regulating glutathione metabolism. The three pathway databases KEGG (https://www.genome.jp/kegg (accessed on 24 April 2022)), Reactome (https://reactome.org (accessed on 27 April 2022)), and WikiPathways (https://www.wikipathways.org (accessed on 24 April 2022)) were used to select genes representing each of the above groups. In particular, pathways “Glutathione metabolism” (KEGG, WikiPathways), “Cysteine and methionine metabolism” (KEGG), “Glutathione synthesis and recycling” (Reactome), “Gamma-glutamyl cycle for the biosynthesis and degradation of glutathione, including diseases“ (WikiPathways) were used to select genes regulating glutathione metabolism. “Protein processing in endoplasmic reticulum “(KEGG) and “Unfolded Protein Response” (Reactome, WikiPathways) pathways were used for selection of genes encoding molecular chaperones or involved in protein folding and unfolded protein response. The criterion for gene selection was the representation of a gene in at least two of the three pathway databases. The CTH gene was added to the list of glutathione metabolism genes because it encodes cystathionine gamma-lyase, which catalyzes the last step in the trans-sulfuration pathway from L-methionine to L-cysteine, which is utilized by cells for biosynthesis of glutathione (https://www.uniprot.org/, accessed on 3 July 2023). The DNAJC3 [45], EDEM2 [16], and GFER [89] genes were also selected for this study because they were found to be associated with T2D susceptibility in earlier studies. Moreover, the insulin gene (INS) was included in the gene list to investigate the correlation between its expression and the studied genes. As a result, raw expression data on 142 genes were selected from whole transcriptomic datasets 1 and 2, with 86 genes encoding molecular chaperones or those involved in protein folding (PF), 36 genes representing the three arms of the UPR pathway, and 20 genes encoding glutathione metabolism enzymes (GME). The full list of genes selected for the study is presented in Supplementary Table S1.

### 4.3. Data Analysis

The limma R software package version 4.3 was used to investigate differentially expressed genes (DEGs) in pancreatic beta cells of T2D patients and non-diabetic controls from Dataset 1 (GSE20966) [159]. Initially, outlier values in the gene expression data were removed from the entire dataset. Expression data were log2-transformed and normalized. Dataset 2 (GSE81608) was processed with the same approach using GREIN (http://www.ilincs.org/apps/grein/, accessed on 6 June 2023), an interactive web platform for exploring and analyzing GEO RNA-seq data to obtain differentially expressed genes [160]. The log fold-change (LogFC) measure, the log-ratio of a gene’s expression values in two analyzed samples, was used to describe the quantity of changes in gene expression existing between T2D patients and non-diabetic controls. The false discovery rate (FDR) to control for multiple comparisons (FDR < 0.05) was calculated by FDR online calculator (https://www.sdmproject.com/utilities/?show=FDR, accessed on 6 June 2023) to identify differentially expressed genes among the 143 selected genes. The expression levels of 143 genes were extracted from both the transcriptome datasets for further statistical analysis using STATISTICA Ultimate version 13.0 (TIBCO, Palo Alto, CA, USA). Pearson’s correlation coefficients were calculated to measure the relationships between expression levels of the genes separately in diabetics and non-diabetics for each dataset. Hierarchical cluster analysis of correlation matrices (Ward’s method) was applied to visualize the groups of co-expressed genes. Linear regression analysis was used to evaluate whether the expression of genes for glutathione metabolism has an impact on the expression levels of genes regulating protein folding and unfolded protein response in pancreatic beta cells of patients with type 2 diabetes and non-diabetic individuals. 

## 5. Conclusions

The accumulation of misfolded and unfolded proteins in the endoplasmic reticulum is known to cause ER stress by activating a signaling pathway known as the unfolded protein response [161,162], focusing on restoring homeostasis by inhibiting the transcription of genes, enhancing the folding capacity of the ER, and degrading unfolded proteins [163]. However, chronic activation of UPR makes ER fail to recover protein homeostasis, resulting in cellular dysfunction and apoptosis [164]. Numerous studies have demonstrated that chronic ER stress and associated UPR are characteristic features of type 2 diabetes, and accumulation of misfolded proinsulin molecules in β-cells is detected early in prediabetes and subsequently exacerbated, leading to apoptosis [19,20,21]. The primary mechanisms underlying ER stress activation and subsequent induction of beta cell apoptosis in type 2 diabetes mellitus remain unknown.

The present study is the first to discover differentially expressed genes encoding enzymes for glutathione metabolism, molecular chaperones, and the UPR pathway in pancreatic beta cells of patients with type 2 diabetes. Although differentially expressed genes varied across the studied transcriptome datasets, the common findings were a decrease in the expression levels of two genes for glutathione metabolism, *GCLC* and *PGD*, as well as differentially expressed genes regulating protein folding, such as *POM121C* and *RPS19BP1*. Three other DEGs regulating glutathione metabolism, *ANPEP* (Dataset 1) and *IDH2* and *CTH* (Dataset 2), were observed in pancreatic beta cells of diabetic patients. Furthermore, beta cells of T2D patients showed a dataset-specific gene signature for DEGs involved in the regulation of protein folding. In particular, beta cells of diabetics exhibited decreased expression of genes encoding molecular chaperones belonging to the heat shock families Hsp70 (*HSPA1B*, *HSPA7*, and *HSPA8*) and Hsp90 (*HSP90AA1* and *HSP90AB1*) as well as co-chaperones (*BAG3* and *ST13*) and nucleoporins (*NDC1*, *NUP160*, and *POM121C*). T2D samples also exhibit DEGs regulating the unfolded protein response: decreased expression of *BID*, a protein that induces caspase and apoptosis pathways (Dataset 2), and increased expression of *CREB3L4* and *ERP27* (Dataset 1). The expression of molecular chaperones and genes involved in the UPR pathway in T2D patients was influenced by the expression of genes responsible for de novo biosynthesis of glutathione, *GCLC*, *GCLM*, and *GSS*, which supports the hypothesis that dysregulation of glutathione biosynthesis might be a key condition responsible for the impaired folding of proinsulin, triggering an unfolded protein response, and ultimately leading to β-cell apoptosis and type 2 diabetes [16]. Undoubtedly, these findings warrant further investigation. The discovery of gene networks involved in the coordinated regulation of gene expression modulating glutathione metabolism, protein folding, and unfolded protein response will allow for a better understanding of the molecular mechanisms governing redox homeostasis in type 2 diabetes, as well as unravel the cross-talk between impaired glutathione metabolism and proteostasis, linking them to well-recognized pathological processes underlying disease development and progression.

## Figures and Tables

**Table 1:** Differentially expressed genes in pancreatic β-cells in patients with type 2 diabetes. (Dataset 1, microarray data).

Gene	Mean (Standard Deviation)		logFC ^1^	*p*-Value ^1^	FDR
	T2D Patients	Non-Diabetics			
Genes for glutathione metabolism					
*ANPEP*	6.86 (0.92)	4.93 (0.93)	1.325	**0.00028**	**0.019**
*GCLC*	7.46 (0.23)	7.81 (0.23)	−0.664	**0.022**	0.218
*G6PD*	5.98 (0.31)	5.70 (0.23)	0.337	**0.037**	0.263
*PGD*	5.62 (0.36)	5.98 (0.21)	−0.262	**0.020**	0.218
Genes regulating protein folding					
*BAG3*	9.26 0.37	9.99 (0.45)	−0.905	**0.0003**	**0.019**
*HSPA7*	4.76 (0.35)	5.05 (0.40)	−0.504	**0.022**	0.218
*HSPB2*	4.42 (1.12)	4.72 (0.69)	−0.962	**0.04**	0.272
*RLN1*	5.73 (0.45)	6.27 (0.37)	−0.763	**0.0018**	**0.037**
*RPS19BP1*	4.72 (0.67)	3.35 (0.56)	1.314	**0.0015**	**0.037**
*NDC1*	5.98 (0.45)	6.55 (0.22)	−0.689	**0.0016**	**0.037**
*RAE1*	7.14 (0.73)	6.49 (0.96)	0.926	**0.018**	0.218
*MTOR*	5.14 (0.38)	5.30 (0.34)	0.399	**0.018**	0.218
*POM121C*	5.35 (0.54)	4.86 (0.36)	0.597	**0.034**	0.263
*TNFRSF21*	10.66 (0.32)	11.01 (0.18)	−0.772	**0.026**	0.234
*GFER*	7.12 (0.39)	6.63 (0.35)	0.317	**0.023**	0.218
Genes of the unfolded protein response pathway					
*CREB3L2*	11.50 (0.29)	11.70 (0.18)	1.392	**0.007**	0.126
*CREB3L4*	3.02 (0.47)	2.53 (0.34)	0.770	**0.0004**	**0.019**
*SSR1*	5.20 (0.33)	5.34 (0.67)	−0.559	**0.008**	0.132
*DNAJB11*	7.28 (0.38)	7.65 (0.35)	−0.482	**0.035**	0.263
*HERPUD1*	10.71 (0.27)	11.00 (0.31)	−0.388	**0.036**	0.263
*ERP27*	7.33 (1.17)	6.49 (0.38)	1.697	**0.0005**	**0.019**

^1^ Calculated for log10-normalized expression levels; FDR, false discovery rate-adjusted *p*-value (Q-values). Bold depicts significant *p* or Q values.

**Table 2:** Differentially expressed genes in pancreatic β-cells in patients with type 2 diabetes (Dataset 2, RNA sequencing data).

Gene	Median (Q1-Q3 Interquartile Range) ^1^		logFC ^2^	*p*-Value ^2^	FDR
	T2D Patients	Non-Diabetics			
Genes for glutathione metabolism					
*GCLC*	31,392.0 (15,207.1–36,668.1)	217.4 (65.4–13,180.9)	−0.658	**0.05**	0.225
*PGD*	13,689.1 (128.2–43,293.5)	129.3 (92.2–169.9)	−1.168	**0.002**	**0.024**
*IDH1*	41,023.9 (118.0–63,847.1)	185.6 (150.1–6897.8)	−0.83	**0.008**	0.063
*IDH2*	8070.4 (6.6–70,496.0)	4.5 (3.1–42.2)	−0.783	**0.006**	**0.05**
*CTH*	44,793.9 (4.5–184,963.7)	40.6 (20.7–76.1)	0.829	**0.003**	**0.033**
Genes regulating protein folding					
*HSP90AB1*	1816.3 (1574.9–2765.8)	2760.6 (2179.6–3129.0)	−0.417	**9.8 × 10^−8^**	**6.04 × 10^−6^**
*HSPA1B*	32,860.4 (27,223.1–42,892.2)	19,142.0 (5.1–76,080.4)	−0.788	**2.8 × 10^−6^**	**0.0001**
*HSP90AA1*	1390.4 (1206.1–1748.1)	1689.4 (1651.8–2289.9)	−0.461	**2.9 × 10^−6^**	**0.0001**
*HSPA8*	1274.0 (1180.6–1350.6)	1579.8 (1223.5–2176.2)	−0.37	**0.001**	**0.015**
*NUP35*	64.0 (30.5–93.5)	55.2 (27.6–73,918.9)	0.794	**0.036**	0.179
*NUP58*	16,049.5 (159.7–30,963.5)	145.1 (110.9–192.4)	0.636	**0.026**	0.142
*NUP107*	155.4 (19.0–52,234.9)	93.1 (52.3–39,162.2)	0.782	**0.013**	0.089
*NUP160*	786.5 (46.5–19,955.0)	50.1 (15.4–24,477.3)	−0.982	**0.002**	**0.027**
*ST13*	405.6 (344.4–480.1)	561.4 (432.5–630.0)	−0.252	**0.05**	0.224
*POM121C*	14,865.3 (27.7–66,158.6)	94.6 (40.2–30,050.1)	−0.669	**0.028**	0.150
*RPS19BP1*	48.0 (13.6–5396.4)	56.9 (32.8–10,441.7)	−0.812	**0.005**	**0.043**
Genes of the unfolded protein response pathway					
*BID*	54,614.2 (48,705.1–81,599.5)	50,649.2 (71.9–151,730.4)	−1.147	**9.0 × 10^−5^**	**0.002**
*DDIT3*	6142.4 (222.0–26,600.4)	280.8 (152.6–477.5)	−0.699	**0.009**	0.069
*HYOU1*	22,083.0 (42.4–105,113.0)	6401.5 (14.1–117,387.1)	−0.609	**0.014**	0.095
*MAPK8*	32.1 (26.6–3174.0)	185.1 (11.7–88,557.5)	−0.947	**0.016**	0.102
*HSPA5*	234.7 (183.3–307.2)	242.0 (140.6–376.1)	−0.438	**0.028**	0.151
*EIF2S3*	571.5 (362.3–727.5)	498.3 (368.9–629.2)	0.23	**0.028**	0.151

^1^ Presented untransformed expression levels; ^2^ Calculated for rank-based inverse-normalized expression levels; FDR, false discovery rate-adjusted *p*-value (Q-values). Bold depicts significant *p* or Q values.

## Data Availability

Data supporting reported results are available upon request.

## Supplementary Materials

The supporting information can be downloaded at: https://www.mdpi.com/article/10.3390/ijms241512059/s1.

## References

[1] Sun H., Saeedi P., Karuranga S., Pinkepank M., Ogurtsova K., Duncan B.B., Stein C., Basit A., Chan J.C.N., Mbanya J.C. (2022). IDF Diabetes Atlas: Global, regional and country-level diabetes prevalence estimates for 2021 and projections for 2045. Diabetes Res. Clin. Pract..

[2] Dedov I., Shestakova M., Benedetti M.M., Simon D., Pakhomov I., Galstyan G. (2016). Prevalence of type 2 diabetes mellitus (T2DM) in the adult Russian population (NATION study). Diabetes Res. Clin. Pract..

[3] Prokopenko I., McCarthy M.I., Lindgren C.M. (2008). Type 2 diabetes: New genes, new understanding. Trends Genet..

[4] DeFronzo R.A., Ferrannini E., Groop L., Henry R.R., Herman W.H., Holst J.J., Hu F.B., Kahn C.R., Raz I., Shulman G.I. (2015). Type 2 diabetes mellitus. Nat. Rev. Dis. Primers.

[5] Porte D., Kahn S.E. (2001). Beta-cell dysfunction and failure in type 2 diabetes: Potential mechanisms. Diabetes.

[6] Weir G.C., Bonner-Weir S. (2004). Five stages of evolving beta-cell dysfunction during progression to diabetes. Diabetes.

[7] Volpe C.M.O., Villar-Delfino P.H., Dos Anjos P.M.F., Nogueira-Machado J.A. (2018). Cellular death, reactive oxygen species (ROS) and diabetic complications. Cell Death Dis..

[8] Drews G., Krippeit-Drews P., Düfer M. (2010). Oxidative stress and beta-cell dysfunction. Pflug. Arch..

[9] Leenders F., Groen N., de Graaf N., Engelse M.A., Rabelink T.J., de Koning E.J.P., Carlotti F. (2021). Oxidative Stress Leads to β-Cell Dysfunction Through Loss of β-Cell Identity. Front. Immunol..

[10] Powell L.A., Warpeha K.M., Xu W., Walker B., Trimble E.R. (2004). High glucose decreases intracellular glutathione concentrations and upregulates inducible nitric oxide synthase gene expression in intestinal epithelial cells. J. Mol. Endocrinol..

[11] Lutchmansingh F.K., Hsu J.W., Bennett F.I., Badaloo A.V., McFarlane-Anderson N., Gordon-Strachan G.M., Wright-Pascoe R.A., Jahoor F., Boyne M.S. (2018). Glutathione metabolism in type 2 diabetes and its relationship with microvascular complications and glycemia. PLoS ONE.

[12] Sekhar R.V., McKay S.V., Patel S.G., Guthikonda A.P., Reddy V.T., Balasubramanyam A., Jahoor F. (2011). Glutathione synthesis is diminished in patients with uncontrolled diabetes and restored by dietary supplementation with cysteine and glycine. Diabetes Care.

[13] Tan K.S., Lee K.O., Low K.C., Gamage A.M., Liu Y., Tan G.Y., Koh H.Q., Alonso S., Gan Y.H. (2012). Glutathione deficiency in type 2 diabetes impairs cytokine responses and control of intracellular bacteria. J. Clin. Investig..

[14] Furfaro A.L., Nitti M., Marengo B., Domenicotti C., Cottalasso D., Marinari U.M., Pronzato M.A., Traverso N. (2012). Impaired synthesis contributes to diabetes-induced decrease in liver glutathione. Int. J. Mol. Med..

[15] Zhang J., An H., Ni K., Chen B., Li H., Li Y., Sheng G., Zhou C., Xie M., Chen S. (2019). Glutathione prevents chronic oscillating glucose intake-induced β-cell dedifferentiation and failure. Cell Death Dis..

[16] Azarova I., Klyosova E., Polonikov A. (2021). The Link between Type 2 Diabetes Mellitus and the Polymorphisms of Glutathione-Metabolizing Genes Suggests a New Hypothesis Explaining Disease Initiation and Progression. Life.

[17] Eizirik D.L., Cardozo A.K., Cnop M. (2008). The role for endoplasmic reticulum stress in diabetes mellitus. Endocr. Rev..

[18] Eizirik D.L., Pasquali L., Cnop M. (2020). Pancreatic β-cells in type 1 and type 2 diabetes mellitus: Different pathways to failure. Nat. Rev. Endocrinol..

[19] Sun J., Cui J., He Q., Chen Z., Arvan P., Liu M. (2015). Proinsulin misfolding and endoplasmic reticulum stress during the development and progression of diabetes. Mol. Asp. Med..

[20] Haataja L., Manickam N., Soliman A., Tsai B., Liu M., Arvan P. (2016). Disulfide Mispairing During Proinsulin Folding in the Endoplasmic Reticulum. Diabetes.

[21] Arunagiri A., Haataja L., Pottekat A., Pamenan F., Kim S., Zeltser L.M., Paton A.W., Paton J.C., Tsai B., Itkin-Ansari P. (2019). Proinsulin misfolding is an early event in the progression to type 2 diabetes. Elife.

[22] Chakravarthi S., Jessop C.E., Bulleid N.J. (2006). The role of glutathione in disulphide bond formation and endoplasmic-reticulum-generated oxidative stress. EMBO Rep..

[23] Okumura M., Saiki M., Yamaguchi H., Hidaka Y. (2011). Acceleration of disulfide-coupled protein folding using glutathione derivatives. FEBS J..

[24] Gough J.D., Williams R.H., Donofrio A.E., Lees W.J. (2002). Folding disulfide-containing proteins faster with an aromatic thiol. J. Am. Chem. Soc..

[25] Neves R.P.P., Fernandes P.A., Ramos M.J. (2017). Mechanistic insights on the reduction of glutathione disulfide by protein disulfide isomerase. Proc. Natl. Acad. Sci. USA.

[26] Cuozzo J.W., Kaiser C.A. (1999). Competition between glutathione and protein thiols for disulphide-bond formation. Nat. Cell Biol..

[27] Delaunay-Moisan A., Ponsero A., Toledano M.B. (2017). Reexamining the Function of Glutathione in Oxidative Protein Folding and Secretion. Antioxid. Redox Signal..

[28] Ruoppolo M., Freedman R.B. (1994). Protein-S-S-glutathione mixed disulfides as models of unfolded proteins. Biochemistry.

[29] Arolas J.L., Aviles F.X., Chang J.Y., Ventura S. (2006). Folding of small disulfide-rich proteins: Clarifying the puzzle. Trends Biochem. Sci..

[30] Tsunoda S., Avezov E., Zyryanova A., Konno T., Mendes-Silva L., Pinho Melo E., Harding H.P., Ron D. (2014). Intact protein folding in the glutathione-depleted endoplasmic reticulum implicates alternative protein thiol reductants. Elife.

[31] Hwang C., Sinskey A.J., Lodish H.F. (1992). Oxidized redox state of glutathione in the endoplasmic reticulum. Science.

[32] Hetz C., Papa F.R. (2018). The Unfolded Protein Response and Cell Fate Control. Mol. Cell.

[33] Back S.H., Kaufman R.J. (2012). Endoplasmic reticulum stress and type 2 diabetes. Annu. Rev. Biochem..

[34] Pandey V.K., Mathur A., Kakkar P. (2019). Emerging role of Unfolded Protein Response (UPR) mediated proteotoxic apoptosis in diabetes. Life Sci..

[35] Mustapha S., Mohammed M., Azemi A.K., Jatau A.I., Shehu A., Mustapha L., Aliyu I.M., Danraka R.N., Amin A., Bala A.A. (2021). Current Status of Endoplasmic Reticulum Stress in Type II Diabetes. Molecules.

[36] Kang Z., Chen F., Wu W., Liu R., Chen T., Xu F. (2022). UPRmt and coordinated UPRER in type 2 diabetes. Front. Cell Dev. Biol..

[37] Shrestha N., De Franco E., Arvan P., Cnop M. (2021). Pathological β-Cell Endoplasmic Reticulum Stress in Type 2 Diabetes: Current Evidence. Front. Endocrinol..

[38] Azarova I., Klyosova E., Lazarenko V., Konoplya A., Polonikov A. (2020). Genetic variants in glutamate cysteine ligase confer protection against type 2 diabetes. Mol. Biol. Rep..

[39] Azarova I.E., Klyosova E.Y., Polonikov A.V. (2021). Polymorphic variants of glutathione reductase—New genetic markers of predisposition to type 2 diabetes mellitus. Ter. Arkh..

[40] Azarova I.E., Klyosova E.Y., Churilin M.I., Samgina T.A., Konoplya A.I., Polonikov A.V. (2020). Genetic and biochemical investigation of the gamma-glutamylcyclotransferase role in predisposition to type 2 diabetes mellitus. Ecol. Genet..

[41] Mastana S.S., Kaur A., Hale R., Lindley M.R. (2013). Influence of glutathione S-transferase polymorphisms (GSTT1, GSTM1, GSTP1) on type-2 diabetes mellitus (T2D) risk in an endogamous population from north India. Mol. Biol. Rep..

[42] Azarova I., Bushueva O., Konoplya A., Polonikov A. (2018). Glutathione S-transferase genes and the risk of type 2 diabetes mellitus: Role of sexual dimorphism, gene-gene and gene-smoking interactions in disease susceptibility. J. Diabetes.

[43] Azarova I., Polonikov A., Klyosova E. (2023). Molecular Genetics of Abnormal Redox Homeostasis in Type 2 Diabetes Mellitus. Int. J. Mol. Sci..

[44] Scheuner D., Kaufman R.J. (2008). The unfolded protein response: A pathway that links insulin demand with beta-cell failure and diabetes. Endocr. Rev..

[45] Lytrivi M., Senée V., Salpea P., Fantuzzi F., Philippi A., Abdulkarim B., Sawatani T., Marín-Cañas S., Pachera N., Degavre A. (2021). DNAJC3 deficiency induces β-cell mitochondrial apoptosis and causes syndromic young-onset diabetes. Eur. J. Endocrinol..

[46] Klyosova E.Y. (2022). Genetic variation of ERN1 and susceptibility to type 2 diabetes. Res. Results Biomed..

[47] Xu B., Allard C., Alvarez-Mercado A.I., Fuselier T., Kim J.H., Coons L.A., Hewitt S.C., Urano F., Korach K.S., Levin E.R. (2018). Estrogens Promote Misfolded Proinsulin Degradation to Protect Insulin Production and Delay Diabetes. Cell Rep..

[48] Costes S., Langen R., Gurlo T., Matveyenko A.V., Butler P.C. (2013). β-Cell failure in type 2 diabetes: A case of asking too much of too few?. Diabetes.

[49] Senft D., Ronai Z.A. (2015). UPR, autophagy, and mitochondria crosstalk underlies the ER stress response. Trends Biochem. Sci..

[50] Ozcan U., Yilmaz E., Ozcan L., Furuhashi M., Vaillancourt E., Smith R.O., Görgün C.Z., Hotamisligil G.S. (2006). Chemical chaperones reduce ER stress and restore glucose homeostasis in a mouse model of type 2 diabetes. Science.

[51] Takayama S., Xie Z., Reed J.C. (1999). An evolutionarily conserved family of Hsp70/Hsc70 molecular chaperone regulators. J. Biol. Chem..

[52] Rosati A., Graziano V., De Laurenzi V., Pascale M., Turco M.C. (2011). BAG3: A multifaceted protein that regulates major cell pathways. Cell Death Dis..

[53] Carra S., Seguin S.J., Lambert H., Landry J. (2008). HspB8 chaperone activity toward poly(Q)-containing proteins depends on its association with Bag3, a stimulator of macroautophagy. J. Biol. Chem..

[54] Ulbricht A., Höhfeld J. (2013). Tension-induced autophagy: May the chaperone be with you. Autophagy.

[55] Iorio V., Festa M., Rosati A., Hahne M., Tiberti C., Capunzo M., De Laurenzi V., Turco M.C. (2015). BAG3 regulates formation of the SNARE complex and insulin secretion. Cell Death Dis..

[56] Mayer M.P., Bukau B. (2005). Hsp70 chaperones: Cellular functions and molecular mechanism. Cell Mol. Life Sci..

[57] Nakhjavani M., Morteza A., Khajeali L., Esteghamati A., Khalilzadeh O., Asgarani F., Outeiro T.F. (2010). Increased serum HSP70 levels are associated with the duration of diabetes. Cell Stress Chaperones.

[58] Radons J. (2016). The human HSP70 family of chaperones: Where do we stand?. Cell Stress Chaperones.

[59] Mir K.A., Pugazhendhi S., Paul M.J., Nair A., Ramakrishna B.S. (2009). Heat-shock protein 70 gene polymorphism is associated with the severity of diabetic foot ulcer and the outcome of surgical treatment. Br. J. Surg..

[60] Gombos T., Förhécz Z., Pozsonyi Z., Jánoskuti L., Prohászka Z. (2008). Interaction of serum 70-kDa heat shock protein levels and HspA1B (+1267) gene polymorphism with disease severity in patients with chronic heart failure. Cell Stress Chaperones.

[61] Zuehlke A.D., Beebe K., Neckers L., Prince T. (2015). Regulation and function of the human HSP90AA1 gene. Gene.

[62] Lee J.H., Gao J., Kosinski P.A., Elliman S.J., Hughes T.E., Gromada J., Kemp D.M. (2013). Heat shock protein 90 (HSP90) inhibitors activate the heat shock factor 1 (HSF1) stress response pathway and improve glucose regulation in diabetic mice. Biochem. Biophys. Res. Commun..

[63] Jing E., Sundararajan P., Majumdar I.D., Hazarika S., Fowler S., Szeto A., Gesta S., Mendez A.J., Vishnudas V.K., Sarangarajan R. (2018). Hsp90β knockdown in DIO mice reverses insulin resistance and improves glucose tolerance. Nutr. Metab..

[64] Ann S.J., Bang H., Lee C.J., Oh J., Park S., Kang S.M., Choi J.K., Lee S.H. (2021). LncRNA HSPA7 in human atherosclerotic plaques sponges miR-223 and promotes the proinflammatory vascular smooth muscle cell transition. Exp. Mol. Med..

[65] Chiva-Blanch G., Peña E., Cubedo J., García-Arguinzonis M., Pané A., Gil P.A., Perez A., Ortega E., Padró T., Badimon L. (2021). Molecular mapping of platelet hyperreactivity in diabetes: The stress proteins complex HSPA8/Hsp90/CSK2α and platelet aggregation in diabetic and normal platelets. Transl. Res..

[66] Suzuki A., Sugiyama Y., Hayashi Y., Nyu-i N., Yoshida M., Nonaka I., Ishiura S., Arahata K., Ohno S. (1998). MKBP, a novel member of the small heat shock protein family, binds and activates the myotonic dystrophy protein kinase. J. Cell Biol..

[67] Ishiwata T., Orosz A., Wang X., Mustafi S.B., Pratt G.W., Christians E.S., Boudina S., Abel E.D., Benjamin I.J. (2012). HSPB2 is dispensable for the cardiac hypertrophic response but reduces mitochondrial energetics following pressure overload in mice. PLoS ONE.

[68] Toft D.J., Fuller M., Schipma M., Chen F., Cryns V.L., Layden B.T. (2016). αB-crystallin and HspB2 deficiency is protective from diet-induced glucose intolerance. Genom. Data.

[69] Mansfeld J., Güttinger S., Hawryluk-Gara L.A., Panté N., Mall M., Galy V., Haselmann U., Mühlhäusser P., Wozniak R.W., Mattaj I.W. (2006). The conserved transmembrane nucleoporin NDC1 is required for nuclear pore complex assembly in vertebrate cells. Mol. Cell.

[70] Kabachinski G., Schwartz T.U. (2015). The nuclear pore complex—Structure and function at a glance. J. Cell Sci..

[71] Bindra D., Mishra R.K. (2021). In Pursuit of Distinctiveness: Transmembrane Nucleoporins and Their Disease Associations. Front. Oncol..

[72] Hawryluk-Gara L.A., Shibuya E.K., Wozniak R.W. (2005). Vertebrate Nup53 interacts with the nuclear lamina and is required for the assembly of a Nup93-containing complex. Mol. Biol. Cell.

[73] Ferrández-Ayela A., Alonso-Peral M.M., Sánchez-García A.B., Micol-Ponce R., Pérez-Pérez J.M., Micol J.L., Ponce M.R. (2013). Arabidopsis TRANSCURVATA1 encodes NUP58, a component of the nucleopore central channel. PLoS ONE.

[74] Boehmer T., Enninga J., Dales S., Blobel G., Zhong H. (2003). Depletion of a single nucleoporin, Nup107, prevents the assembly of a subset of nucleoporins into the nuclear pore complex. Proc. Natl. Acad. Sci. USA.

[75] Vasu S., Shah S., Orjalo A., Park M., Fischer W.H., Forbes D.J. (2001). Novel vertebrate nucleoporins Nup133 and Nup160 play a role in mRNA export. J. Cell Biol..

[76] Xie J., Yuan Y., Yao G., Chen Z., Yu W., Zhu Q. (2021). Nucleoporin 160 (NUP160) inhibition alleviates diabetic nephropathy by activating autophagy. Bioengineered.

[77] Lundbäck V., Kulyte A., Strawbridge R.J., Ryden M., Arner P., Marcus C., Dahlman I. (2018). FAM13A and POM121C are candidate genes for fasting insulin: Functional follow-up analysis of a genome-wide association study. Diabetologia.

[78] Pan G., Bauer J.H., Haridas V., Wang S., Liu D., Yu G., Vincenz C., Aggarwal B.B., Ni J., Dixit V.M. (1998). Identification and functional characterization of DR6, a novel death domain-containing TNF receptor. FEBS Lett..

[79] Hsu P.C., Huang J.C., Tsai W.C., Hung W.W., Chang W.A., Wu L.Y., Chang C.Y., Tsai Y.C., Hsu Y.L. (2022). Tumor Necrosis Factor Receptor Superfamily Member 21 Induces Endothelial-Mesenchymal Transition in Coronary Artery Endothelium of Type 2 Diabetes Mellitus. Biomedicines.

[80] Niewczas M.A., Pavkov M.E., Skupien J., Smiles A., Md Dom Z.I., Wilson J.M., Park J., Nair V., Schlafly A., Saulnier P.J. (2019). A signature of circulating inflammatory proteins and development of end-stage renal disease in diabetes. Nat. Med..

[81] Ren Y., Seo H.S., Blobel G., Hoelz A. (2010). Structural and functional analysis of the interaction between the nucleoporin Nup98 and the mRNA export factor Rae1. Proc. Natl. Acad. Sci. USA.

[82] Mao Z., Zhang W. (2018). Role of mTOR in Glucose and Lipid Metabolism. Int. J. Mol. Sci..

[83] Guillén C., Benito M. (2018). mTORC1 Overactivation as a Key Aging Factor in the Progression to Type 2 Diabetes Mellitus. Front. Endocrinol..

[84] Kim E.J., Kho J.H., Kang M.R., Um S.J. (2007). Active regulator of SIRT1 cooperates with SIRT1 and facilitates suppression of p53 activity. Mol. Cell.

[85] Banks A.S., Kon N., Knight C., Matsumoto M., Gutiérrez-Juárez R., Rossetti L., Gu W., Accili D. (2008). SirT1 gain of function increases energy efficiency and prevents diabetes in mice. Cell Metab..

[86] Fischer M., Riemer J. (2013). The mitochondrial disulfide relay system: Roles in oxidative protein folding and beyond. Int. J. Cell Biol..

[87] Erdogan A.J., Ali M., Habich M., Salscheider S.L., Schu L., Petrungaro C., Thomas L.W., Ashcroft M., Leichert L.I., Roma L.P. (2018). The mitochondrial oxidoreductase CHCHD4 is present in a semi-oxidized state in vivo. Redox Biol..

[88] Al-Habib H., Ashcroft M. (2021). CHCHD4 (MIA40) and the mitochondrial disulfide relay system. Biochem. Soc. Trans..

[89] Klyosova E.Y., Shkurat E.A., Azarova Y.E., Polonikov A.V. (2022). Polymorphism rs1046495 of the GFER Gene as a New Genetic Marker of Preposition to Type 2 Diabetes Mellitus. Bull. Exp. Biol. Med..

[90] Sampieri L., Di Giusto P., Alvarez C. (2019). CREB3 Transcription Factors: ER-Golgi Stress Transducers as Hubs for Cellular Homeostasis. Front. Cell Dev. Biol..

[91] Smith B.S., Diaguarachchige De Silva K.H., Hashemi A., Duncan R.E., Grapentine S., Bakovic M., Lu R. (2022). Transcription factor CREB3 is a potent regulator of high-fat diet-induced obesity and energy metabolism. Int. J. Obes..

[92] Kim T.H., Park J.M., Jo S.H., Kim M.Y., Nojima H., Ahn Y.H. (2015). Effects of low-fat diet and aging on metabolic profiles of Creb3l4 knockout mice. Nutr. Diabetes.

[93] Alanen H.I., Williamson R.A., Howard M.J., Hatahet F.S., Salo K.E., Kauppila A., Kellokumpu S., Ruddock L.W. (2006). ERp27, a new non-catalytic endoplasmic reticulum-located human protein disulfide isomerase family member, interacts with ERp57. J. Biol. Chem..

[94] Marselli L., Thorne J., Dahiya S., Sgroi D.C., Sharma A., Bonner-Weir S., Marchetti P., Weir G.C. (2010). Gene expression profiles of Beta-cell enriched tissue obtained by laser capture microdissection from subjects with type 2 diabetes. PLoS ONE.

[95] Gregory L.C., Ferreira C.B., Young-Baird S.K., Williams H.J., Harakalova M., van Haaften G., Rahman S.A., Gaston-Massuet C., Kelberman D., GOSgene (2019). Impaired EIF2S3 function associated with a novel phenotype of X-linked hypopituitarism with glucose dysregulation. EBioMedicine.

[96] Stanik J., Skopkova M., Stanikova D., Brennerova K., Barak L., Ticha L., Hornova J., Klimes I., Gasperikova D. (2018). Neonatal hypoglycemia, early-onset diabetes and hypopituitarism due to the mutation in EIF2S3 gene causing MEHMO syndrome. Physiol. Res..

[97] Li X., Itani O.A., Haataja L., Dumas K.J., Yang J., Cha J., Flibotte S., Shih H.J., Delaney C.E., Xu J. (2019). Requirement for translocon-associated protein (TRAP) α in insulin biogenesis. Sci. Adv..

[98] Dana R.C., Welch W.J., Deftos L.J. (1990). Heat shock proteins bind calcitonin. Endocrinology.

[99] Kang J.M., Park S., Kim S.J., Kim H., Lee B., Kim J., Park J., Kim S.T., Yang H.K., Kim W.H. (2015). KIAA1324 Suppresses Gastric Cancer Progression by Inhibiting the Oncoprotein GRP78. Cancer Res..

[100] Ng D.T., Watowich S.S., Lamb R.A. (1992). Analysis in vivo of GRP78-BiP/substrate interactions and their role in induction of the GRP78-BiP gene. Mol. Biol. Cell.

[101] Nourbakhsh M., Sharifi R., Heydari N., Nourbakhsh M., Ezzati-Mobasser S., Zarrinnahad H. (2022). Circulating TRB3 and GRP78 levels in type 2 diabetes patients: Crosstalk between glucose homeostasis and endoplasmic reticulum stress. J. Endocrinol. Investig..

[102] Yamagishi N., Ueda T., Mori A., Saito Y., Hatayama T. (2012). Decreased expression of endoplasmic reticulum chaperone GRP78 in liver of diabetic mice. Biochem. Biophys. Res. Commun..

[103] Teodoro-Morrison T., Schuiki I., Zhang L., Belsham D.D., Volchuk A. (2013). GRP78 overproduction in pancreatic beta cells protects against high-fat-diet-induced diabetes in mice. Diabetologia.

[104] Cornec-Le Gall E., Olson R.J., Besse W., Heyer C.M., Gainullin V.G., Smith J.M., Audrézet M.-P., Hopp K., Porath B., Pierre A.M. (2018). Monoallelic Mutations to DNAJB11 Cause Atypical Autosomal-Dominant Polycystic Kidney Disease. Am. J. Hum. Genet..

[105] Shen Y., Hendershot L.M. (2005). ERdj3, a stress-inducible endoplasmic reticulum DnaJ homologue, serves as a cofactor for BiP’s interactions with unfolded substrates. Mol. Biol. Cell.

[106] Yu M., Haslam R.H., Haslam D.B. (2000). HEDJ, an Hsp40 co-chaperone localized to the endoplasmic reticulum of human cells. J. Biol. Chem..

[107] Lu H., Yang Y., Allister E.M., Wijesekara N., Wheeler M.B. (2008). The identification of potential factors associated with the development of type 2 diabetes: A quantitative proteomics approach. Mol. Cell. Proteom..

[108] Schulz J., Avci D., Queisser M.A., Gutschmidt A., Dreher L.S., Fenech E.J., Volkmar N., Hayashi Y., Hoppe T., Christianson J.C. (2017). Conserved cytoplasmic domains promote Hrd1 ubiquitin ligase complex formation for ER-associated degradation (ERAD). J. Cell Sci..

[109] Wong N., Morahan G., Stathopoulos M., Proietto J., Andrikopoulos S. (2013). A novel mechanism regulating insulin secretion involving Herpud1 in mice. Diabetologia.

[110] Renshaw S.A., Dempsey C.E., Barnes F.A., Bagstaff S.M., Dower S.K., Bingle C.D., Whyte M.K. (2004). Three novel Bid proteins generated by alternative splicing of the human Bid gene. J. Biol. Chem..

[111] Zhai D., Luciano F., Zhu X., Guo B., Satterthwait A.C., Reed J.C. (2005). Humanin binds and nullifies Bid activity by blocking its activation of Bax and Bak. J. Biol. Chem..

[112] McKenzie M.D., Carrington E.M., Kaufmann T., Strasser A., Huang D.C., Kay T.W., Allison J., Thomas H.E. (2008). Proapoptotic BH3-only protein Bid is essential for death receptor-induced apoptosis of pancreatic beta-cells. Diabetes.

[113] Oliveira S.J., Pinto J.P., Picarote G., Costa V.M., Carvalho F., Rangel M., de Sousa M., de Almeida S.F. (2009). ER stress-inducible factor CHOP affects the expression of hepcidin by modulating C/EBPalpha activity. PLoS ONE.

[114] Ohoka N., Yoshii S., Hattori T., Onozaki K., Hayashi H. (2005). TRB3, a novel ER stress-inducible gene, is induced via ATF4-CHOP pathway and is involved in cell death. EMBO J..

[115] Yong J., Parekh V.S., Reilly S.M., Nayak J., Chen Z., Lebeaupin C., Jang I., Zhang J., Prakash T.P., Sun H. (2021). Chop/Ddit3 depletion in β cells alleviates ER stress and corrects hepatic steatosis in mice. Sci. Transl. Med..

[116] Oyadomari S., Koizumi A., Takeda K., Gotoh T., Akira S., Araki E., Mori M. (2002). Targeted disruption of the Chop gene delays endoplasmic reticulum stress-mediated diabetes. J. Clin. Investig..

[117] Ozawa K., Kuwabara K., Tamatani M., Takatsuji K., Tsukamoto Y., Kaneda S., Yanagi H., Stern D.M., Eguchi Y., Tsujimoto Y. (1999). 150-kDa oxygen-regulated protein (ORP150) suppresses hypoxia-induced apoptotic cell death. J. Biol. Chem..

[118] Lindenmeyer M.T., Rastaldi M.P., Ikehata M., Neusser M.A., Kretzler M., Cohen C.D., Schlöndorff D. (2008). Proteinuria and hyperglycemia induce endoplasmic reticulum stress. J. Am. Soc. Nephrol..

[119] Wei Y., Sinha S., Levine B. (2008). Dual role of JNK1-mediated phosphorylation of Bcl-2 in autophagy and apoptosis regulation. Autophagy.

[120] Gogg S., Smith U., Jansson P.A. (2009). Increased MAPK activation and impaired insulin signaling in subcutaneous microvascular endothelial cells in type 2 diabetes: The role of endothelin-1. Diabetes.

[121] Bengal E., Aviram S., Hayek T. (2020). p38 MAPK in Glucose Metabolism of Skeletal Muscle: Beneficial or Harmful?. Int. J. Mol. Sci..

[122] Wang X., Khaleque M.A., Zhao M.J., Zhong R., Gaestel M., Calderwood S.K. (2006). Phosphorylation of HSF1 by MAPK-activated protein kinase 2 on serine 121, inhibits transcriptional activity and promotes HSP90 binding. J. Biol. Chem..

[123] Chen Y., Shertzer H.G., Schneider S.N., Nebert D.W., Dalton T.P. (2005). Glutamate cysteine ligase catalysis: Dependence on ATP and modifier subunit for regulation of tissue glutathione levels. J. Biol. Chem..

[124] Locke J.M., Hysenaj G., Wood A.R., Weedon M.N., Harries L.W. (2015). Targeted allelic expression profiling in human islets identifies cis-regulatory effects for multiple variants identified by type 2 diabetes genome-wide association studies. Diabetes.

[125] Ibrahim S., Rashed L. (2007). Estimation of transforming growth factor-beta 1 as a marker of renal injury in type II diabetes mellitus. Saudi Med. J..

[126] Kum J.J.Y., Howlett C.J., Khan Z.A. (2022). Dysregulated transforming growth factor-beta mediates early bone marrow dysfunction in diabetes. Commun. Biol..

[127] Gabrilovac J., Breljak D., Cupić B. (2008). Regulation of aminopeptidase N (EC 3.4.11.2; APN.; CD13) on the HL-60 cell line by TGF-β_1_. Int. Immunopharmacol..

[128] Kooner J.S., Saleheen D., Sim X., Sehmi J., Zhang W., Frossard P., Been L.F., Chia K.S., Dimas A.S., Hassanali N. (2011). Genome-wide association study in individuals of South Asian ancestry identifies six new type 2 diabetes susceptibility loci. Nat. Genet..

[129] Xue A., Wu Y., Zhu Z., Zhang F., Kemper K.E., Zheng Z., Yengo L., Lloyd-Jones L.R., Sidorenko J., Wu Y. (2018). Genome-wide association analyses identify 143 risk variants and putative regulatory mechanisms for type 2 diabetes. Nat. Commun..

[130] Turner A.J., Barrett J., Rawlings N.D., Woessner J.F. (2004). Membrane alanyl aminopeptidase. Handbook of Proteolytic Enzymes.

[131] Uehara N., Fujita M., Shimizu T. (2009). Colorimetric assay of aminopeptidase N activity based on inhibition of the disassembly of gold nano-composites conjugated with a thermo-responsive copolymer. Anal. Sci..

[132] Geisbrecht B.V., Gould S.J. (1999). The human PICD gene encodes a cytoplasmic and peroxisomal NADP(+)-dependent isocitrate dehydrogenase. J. Biol. Chem..

[133] Yu W., Dittenhafer-Reed K.E., Denu J.M. (2012). SIRT3 protein deacetylates isocitrate dehydrogenase 2 (IDH2) and regulates mitochondrial redox status. J. Biol. Chem..

[134] MacDonald M.J., Brown L.J., Longacre M.J., Stoker S.W., Kendrick M.A., Hasan N.M. (2013). Knockdown of both mitochondrial isocitrate dehydrogenase enzymes in pancreatic beta cells inhibits insulin secretion. Biochim. Biophys. Acta.

[135] Dai X., Wang K., Fan J., Liu H., Fan X., Lin Q., Chen Y., Chen H., Li Y., Liu H. (2022). Nrf2 transcriptional upregulation of IDH2 to tune mitochondrial dynamics and rescue angiogenic function of diabetic EPCs. Redox Biol..

[136] Wang H., Xiong Q., He G., Tang J., Sun L., Cheng S., Ke M., Chen S., Hu Y., Feng J. (2023). Hepatic IDH2 regulates glycolysis and gluconeogenesis. Metabolism.

[137] Stanton R.C. (2012). Glucose-6-phosphate dehydrogenase, NADPH, and cell survival. IUBMB Life.

[138] Gansemer E.R., McCommis K.S., Martino M., King-McAlpin A.Q., Potthoff M.J., Finck B.N., Taylor E.B., Rutkowski D.T. (2020). NADPH and Glutathione Redox Link TCA Cycle Activity to Endoplasmic Reticulum Homeostasis. iScience.

[139] Lai Y.K., Lai N.M., Lee S.W. (2017). Glucose-6-phosphate dehydrogenase deficiency and risk of diabetes: A systematic review and meta-analysis. Ann. Hematol..

[140] Zhu W., Lin A., Banerjee R. (2008). Kinetic properties of polymorphic variants and pathogenic mutants in human cystathionine gamma-lyase. Biochemistry.

[141] Krishnan N., Fu C., Pappin D.J., Tonks N.K. (2011). H2S-Induced sulfhydration of the phosphatase PTP1B and its role in the endoplasmic reticulum stress response. Sci. Signal..

[142] Chakravarthi S., Bulleid N.J. (2004). Glutathione is required to regulate the formation of native disulfide bonds within proteins entering the secretory pathway. J. Biol. Chem..

[143] Kwak S.H., Park K.S., Lee K.U., Lee H.K. (2010). Mitochondrial metabolism and diabetes. J. Diabetes Investig..

[144] Marí M., de Gregorio E., de Dios C., Roca-Agujetas V., Cucarull B., Tutusaus A., Morales A., Colell A. (2020). Mitochondrial Glutathione: Recent Insights and Role in Disease. Antioxidants.

[145] Ellgaard L., Ruddock L.W. (2005). The human protein disulphide isomerase family: Substrate interactions and functional properties. EMBO Rep..

[146] Lyles M.M., Gilbert H.F. (1991). Catalysis of the oxidative folding of ribonuclease A by protein disulfide isomerase: Dependence of the rate on the composition of the redox buffer. Biochemistry.

[147] Bocedi A., Cattani G., Gambardella G., Schulte L., Schwalbe H., Ricci G. (2021). Oxidative Folding of Proteins: The “Smoking Gun” of Glutathione. Int. J. Mol. Sci..

[148] Cao S.S., Kaufman R.J. (2014). Endoplasmic reticulum stress and oxidative stress in cell fate decision and human disease. Antioxid. Redox Signal..

[149] Abramov A.Y., Potapova E.V., Dremin V.V., Dunaev A.V. (2020). Interaction of Oxidative Stress and Misfolded Proteins in the Mechanism of Neurodegeneration. Life.

[150] Cullinan S.B., Zhang D., Hannink M., Arvisais E., Kaufman R.J., Diehl J.A. (2003). Nrf2 is a direct PERK substrate and effector of PERK-dependent cell survival. Mol. Cell. Biol..

[151] Harding H.P., Zhang Y., Zeng H., Novoa I., Lu P.D., Calfon M., Sadri N., Yun C., Popko B., Paules R. (2003). An integrated stress response regulates amino acid metabolism and resistance to oxidative stress. Mol. Cell.

[152] Huang H.C., Nguyen T., Pickett C.B. (2001). Regulation of the antioxidant response element by protein kinase C-mediated phosphorylation of NF-E2-related factor 2. Proc. Natl. Acad. Sci. USA.

[153] Pal J., Bera S., Ghosh S.K. (1998). The effect of glutathione upon chaperone activity of alpha-crystallin is probably mediated through target modulation. Ophthalmic Res..

[154] Plaisance V., Brajkovic S., Tenenbaum M., Favre D., Ezanno H., Bonnefond A., Bonner C., Gmyr V., Kerr-Conte J., Gauthier B.R. (2016). Endoplasmic Reticulum Stress Links Oxidative Stress to Impaired Pancreatic Beta-Cell Function Caused by Human Oxidized LDL. PLoS ONE.

[155] Chong W.C., Shastri M.D., Eri R. (2017). Endoplasmic Reticulum Stress and Oxidative Stress: A Vicious Nexus Implicated in Bowel Disease Pathophysiology. Int. J. Mol. Sci..

[156] Ghosh S., Bose S., Gowda S., Mukhopadhyay P. (2019). Biosimilar Insulins—What a Clinician Needs to Know?. Indian J. Endocrinol. Metab..

[157] Porcu E., Sadler M.C., Lepik K., Auwerx C., Wood A.R., Weihs A., Sleiman M.S.B., Ribeiro D.M., Bandinelli S., Tanaka T. (2021). Differentially expressed genes reflect disease-induced rather than disease-causing changes in the transcriptome. Nat. Commun..

[158] Xin Y., Kim J., Okamoto H., Ni M., Wei Y., Adler C., Murphy A.J., Yancopoulos G.D., Lin C., Gromada J. (2016). RNA Sequencing of Single Human Islet Cells Reveals Type 2 Diabetes Genes. Cell Metab..

[159] Ritchie M.E., Phipson B., Wu D., Hu Y., Law C.W., Shi W., Smyth G.K. (2015). Limma powers differential expression analyses for RNA-sequencing and microarray studies. Nucleic Acids Res..

[160] Mahi N.A., Najafabadi M.F., Pilarczyk M., Kouril M., Medvedovic M. (2019). GREIN: An Interactive Web Platform for Re-analyzing GEO RNA-seq Data. Sci. Rep..

[161] Schröder M., Kaufman R.J. (2005). The mammalian unfolded protein response. Annu. Rev. Biochem..

[162] Schröder M. (2008). Endoplasmic reticulum stress responses. Cell Mol. Life Sci..

[163] Yoshida H. (2007). ER stress and diseases. FEBS J..

[164] Kim I., Xu W., Reed J.C. (2008). Cell death and endoplasmic reticulum stress: Disease relevance and therapeutic opportunities. Nat. Rev. Drug Discov..

