# Supplementary material for: Differentially Expressed Genes Regulating Glutathione Metabolism, Protein-Folding, and Unfolded Protein Response in Pancreatic β-Cells in Type 2 Diabetes Mellitus
Source: Int J Mol Sci. 2023 Jul 27;24(15):12059. doi: 10.3390/ijms241512059 (PMC10418503; doi:10.3390/ijms241512059)
Supplement: Supplementary file 1 [file ijms-24-12059-s001.zip › Supplementary Table S3.pdf]

`

Supplementary Table S3 Impact of genes encoding enzymes for glutathione biosynthesis on expression levels of genes for protein folding and unfolded protein response in pancreatic β-cells of non-diabetics and diabetic patients (Dataset 1)

Independent gene (glutathione biosynthesis)	Dependent gene (protein folding /unfolded protein response)	*beta*	Standard error of *beta*	R^2	P-value	FDR adjusted P-value (Q-value)
Diabetic patients						
GCLC	*EP300*	-0.872	0.173	0.761	**0.001**	0.10
GCLC	*HSPA1L*	0.854	0.184	0.729	**0.0016**	0.10
GCLC	*MTOR*	-0.764	0.228	0.584	**0.01**	0.40
GCLC	*CALR*	0.724	0.244	0.524	**0.018**	0.54
GCLC	*DDIT3*	0.647	0.270	0.419	**0.043**	0.73
GCLC	*CREBRF*	-0.664	0.264	0.441	**0.036**	0.71
GCLC	*TM7SF4*	0.699	0.253	0.489	**0.024**	0.57
GCLM	*ATR*	-0.652	0.268	0.426	**0.04**	0.99
GCLM	*NUP153*	0.723	0.244	0.522	**0.018**	0.99
GCLM	*RAE1*	-0.697	0.254	0.485	**0.025**	0.99
GSS	*GSK3B*	0.653	0.268	0.427	**0.04**	0.26
GSS	*HSBP1*	0.691	0.256	0.477	**0.026**	0.21
GSS	*HSP90AA1*	0.634	0.274	0.401	**0.049**	0.29
GSS	*HSPA14*	-0.690	0.256	0.476	**0.027**	0.21
GSS	*NUP153*	-0.723	0.244	0.523	**0.018**	0.21
GSS	*NUP214*	0.643	0.271	0.413	**0.045**	0.28
GSS	*NUP58*	-0.677	0.260	0.458	**0.03**	0.22
GSS	*RANBP2*	-0.919	0.139	0.845	**0.00016**	**0.019**
GSS	*CRYAB*	0.664	0.264	0.441	**0.036**	0.25
GSS	*CTDSP2*	0.791	0.216	0.625	**0.006**	0.21
GSS	*ATF4*	0.710	0.249	0.504	**0.021**	0.21
GSS	*BID*	0.695	0.254	0.483	**0.026**	0.21
GSS	*CALR*	0.716	0.247	0.513	**0.019**	0.21
GSS	*DDIT3*	0.701	0.252	0.491	**0.02**	0.21
GSS	*CKAP4*	-0.712	0.248	0.507	**0.02**	0.21
GSS	*CREB3L2*	-0.771	0.225	0.595	**0.009**	0.21
GSS	*DNAJB11*	0.719	0.246	0.516	**0.019**	0.21
GSS	*EDEM1*	-0.879	0.169	0.773	**0.0008**	**0.047**
GSS	*EIF2AK3*	-0.752	0.233	0.566	**0.01**	0.21
GSS	*XBP1*	0.702	0.252	0.493	**0.02**	0.21
Non-diabetic patients						
GCLM	*BAG1*	0.733	0.241	0.537	**0.015**	0.13
GCLM	*BAG2*	0.841	0.191	0.707	**0.002**	**0.046**
GCLM	*DNAJB6*	0.863	0.178	0.745	**0.001**	**0.048**
GCLM	*DNAJC7*	0.670	0.262	0.449	**0.03**	0.18
GCLM	*HSPA14*	0.825	0.199	0.681	**0.003**	0.059
GCLM	*HSPA8*	0.821	0.202	0.674	**0.0036**	0.059
GCLM	*HSPA9*	0.720	0.245	0.518	**0.02**	0.15
GCLM	*NDC1*	0.669	0.263	0.447	**0.03**	0.18
GCLM	*NDC1*	0.669	0.263	0.447	**0.03**	0.18
GCLM	*NUP153*	0.763	0.228	0.583	**0.01**	0.09
GCLM	*NUP160*	0.849	0.187	0.722	**0.0018**	**0.047**
GCLM	*NUP205*	0.810	0.207	0.656	**0.004**	0.059
GCLM	*NUP214*	-0.876	0.171	0.767	**0.0009**	**0.048**
GCLM	*NUP54*	0.765	0.228	0.585	**0.0099**	0.09
GCLM	*NUP58*	0.761	0.230	0.578	**0.01**	0.09
GCLM	*POM121C*	0.651	0.268	0.424	**0.04**	0.20
GCLM	*PTGES3*	0.782	0.221	0.611	**0.008**	0.09

`

GCLM	*SIRT1*	0.783	0.220	0.613	**0.007**	0.09
GCLM	*ST13*	0.644	0.270	0.415	**0.04**	0.20
GCLM	*EEF1A1*	0.647	0.270	0.418	**0.04**	0.20
GCLM	*ASNS*	0.683	0.258	0.466	**0.029**	0.18
GCLM	*EDEM2*	-0.645	0.270	0.416	**0.04**	0.20
GCLM	*EIF2S2*	0.730	0.242	0.532	**0.016**	0.13
GCLM	*PDIA6*	0.675	0.261	0.456	**0.03**	0.18
GCLM	*SSR1*	0.843	0.190	0.711	**0.002**	**0.048**
GSS	*BAG1*	-0.771	0.225	0.595	**0.009**	0.15
GSS	*BAG2*	-0.703	0.251	0.494	**0.02**	0.20
GSS	*BAG4*	0.763	0.229	0.582	**0.01**	0.15
GSS	*DNAJB6*	-0.755	0.232	0.570	**0.01**	0.15
GSS	*HSPA14*	-0.722	0.244	0.522	**0.018**	0.20
GSS	*NDC1*	-0.709	0.249	0.503	**0.02**	0.20
GSS	*NUP133*	-0.699	0.253	0.489	**0.02**	0.20
GSS	*NUP153*	-0.654	0.267	0.428	**0.04**	0.28
GSS	*NUP160*	-0.666	0.264	0.444	**0.035**	0.26
GSS	*NUP210*	0.782	0.220	0.611	**0.007**	0.15
GSS	*NUP214*	0.637	0.273	0.405	**0.047**	0.29
GSS	*NUP37*	-0.686	0.257	0.471	**0.028**	0.26
GSS	*NUP50*	0.633	0.274	0.401	**0.049**	0.29
GSS	*NUP54*	-0.670	0.263	0.448	**0.03**	0.26
GSS	*NUP88*	-0.792	0.216	0.627	**0.006**	0.15
GSS	*RANBP2*	-0.780	0.221	0.608	**0.008**	0.15
GSS	*RPA1*	-0.641	0.271	0.411	**0.045**	0.29
GSS	*ST13*	-0.792	0.216	0.627	**0.006**	0.15
GSS	*TPR*	-0.844	0.190	0.712	**0.002**	0.15
GSS	*CRYAB*	-0.667	0.264	0.444	**0.035**	0.26

Beta, the regression coefficient; R^2, R-squared; FDR adjusted P-value was calculated with FDR online calculator (https://www.sdmproject.com/utilities/?show=FDR, assessed 10.06.2023);
Bold depicts statistically significant P- and Q-values.
