# Supplementary material for: Differentially Expressed Genes Regulating Glutathione Metabolism, Protein-Folding, and Unfolded Protein Response in Pancreatic β-Cells in Type 2 Diabetes Mellitus
Source: Int J Mol Sci. 2023 Jul 27;24(15):12059. doi: 10.3390/ijms241512059 (PMC10418503; doi:10.3390/ijms241512059)
Supplement: Supplementary file 1 [file ijms-24-12059-s001.zip › Supplementary Table S4.pdf]

`

Supplementary Table S4 Impact of genes encoding enzymes for glutathione biosynthesis on expression levels of genes for protein folding and unfolded protein response in pancreatic β-cells of non-diabetics and diabetic patients (Dataset 2)

Independent gene (glutathione biosynthesis)	Dependent gene (protein folding /unfolded protein response)	*beta*	Standard error of *beta*	R^2	P-value	FDR adjusted P-value (Q-value)
Diabetic patients						
GCLC	*DNAJA2*	-0.825	0.283	0.681	**0.04**	0.34
GCLC	*HSPB1*	-0.817	0.288	0.668	**0.047**	0.36
GCLC	*NUP153*	0.982	0.094	0.964	**0.0005**	**0.047**
GCLC	*NUP58*	0.857	0.258	0.734	**0.029**	0.27
GCLC	*TPR*	0.956	0.147	0.914	**0.0028**	0.11
GCLC	*DNAJB1*	-0.815	0.289	0.665	**0.048**	0.36
GCLM	*POM121C*	0.992	0.063	0.984	**0.0001**	**0.01**
GCLM	*RPA2*	0.883	0.235	0.780	**0.019**	0.18
GCLM	*CREB3L4*	0.881	0.236	0.776	**0.02**	0.18
GCLM	*EIF2S1*	0.906	0.211	0.821	**0.01**	0.18
GCLM	*PPP1R15A*	0.942	0.167	0.888	**0.004**	0.16
GSS	*DNAJC7*	0.877	0.241	0.769	**0.02**	0.17
GSS	*HSPA1L*	-0.998	0.048	0.995	**0.002**	0.08
GSS	*NUP160*	0.906	0.211	0.822	**0.01**	0.17
GSS	*RPA2*	0.872	0.245	0.760	**0.02**	0.17
GSS	*TPR*	-0.887	0.231	0.787	**0.018**	0.17
GSS	*DNAJB1*	0.969	0.123	0.939	**0.001**	0.06
Non-diabetic patients						
GCLC	*HSP90B1*	0.586	0.256	0.343	**0.045**	0.27
GCLC	*GFER*	-0.756	0.218	0.572	**0.007**	0.16
GCLC	*DNAJC7*	0.595	0.254	0.354	**0.04**	0.25
GCLC	*HSBP1*	0.652	0.240	0.425	**0.02**	0.17
GCLC	*HSP90AA1*	0.663	0.237	0.439	**0.018**	0.17
GCLC	*HSPA9*	0.605	0.252	0.366	**0.037**	0.24
GCLC	*ATF4*	0.662	0.237	0.438	**0.019**	0.17
GCLC	*DDIT3*	0.668	0.235	0.446	**0.018**	0.17
GCLC	*EDEM1*	0.804	0.188	0.646	**0.0016**	0.09
GCLM	*PPP1R15A*	-0.585	0.257	0.342	**0.046**	0.29
GCLM	*SYVN1*	-0.593	-0.593	0.352	**0.04**	0.26
GCLM	*BAG1*	0.750	0.209	0.562	**0.005**	0.21
GCLM	*BAG2*	-0.624	0.247	0.389	**0.03**	0.22
GCLM	*DNAJC2*	-0.615	0.249	0.378	**0.03**	0.22
GCLM	*FKBP4*	-0.589	0.255	0.347	**0.04**	0.26
GCLM	*HSPB1*	-0.697	0.239	0.485	**0.017**	0.21
GCLM	*NUP43*	0.617	0.249	0.380	**0.03**	0.22
GCLM	*POM121*	0.706	0.224	0.498	**0.01**	0.21
GSS	*AAAS*	0.594	0.254	0.352	**0.04**	0.40
GSS	*NUP188*	-0.585	0.257	0.342	**0.045**	0.41
GSS	*HERPUD1*	0.705	0.224	0.497	**0.01**	0.25

Beta, the regression coefficient; R^2, R-squared; FDR adjusted P-value was calculated with FDR online calculator (https://www.sdmproject.com/utilities/?show=FDR, assessed 10.06.2023);
Bold depicts statistically significant P- and Q-values.
